# Roux-en-Y Gastric Bypass versus Sleeve Gastrectomy for Cardiometabolic Outcomes: A Systematic Review and Meta-analysis of Randomized Controlled Trials

**DOI:** 10.1007/s11695-026-08664-8

**Published:** 2026-04-29

**Authors:** Felipe Pereira Garrido Pazos, Rafael Matos Vieira Gordilho, Eduardo de Oliveira Novelli, Luiz Guilherme Lima Santana, Maria da Conceição Cavalcanti de Figueiredo Vilaboim, Breno Santos Matos de Magalhães, Maria Luisa França Lessa, Pedro Henrique Matos Oliveira, Marcelo Falcão

**Affiliations:** 1 Department of Medicine, Bahiana School of Medicine and Public Health, Salvador, Brazil; 2 Falcão Institute of Endoscopy and Surgery, Salvador, Brazil

## Abstract

This systematic review and meta-analysis of randomized controlled trials compared Roux-en-Y gastric bypass (RYGB) and sleeve gastrectomy (SG) regarding cardiometabolic outcomes in adults with obesity. Twenty-four trials including 2,890 participants were analyzed across short (1 year), mid- (between 1 and 10 years), and long-term (10 years) follow-up. In the short term, RYGB was associated with greater improvements in lipid profile, glycemic control, and C-reactive protein, as well as higher remission rates of type 2 diabetes mellitus and dyslipidemia. These metabolic advantages persisted in the mid term, particularly for lipid outcomes. At long-term follow-up, no differences between procedures were observed. Overall, RYGB appears to provide greater short- and mid-term cardiometabolic benefits compared with SG, while long-term outcomes are similar, supporting individualized procedure selection.

**Supplementary Information:**

The online version contains supplementary material available at 10.1007/s11695-026-08664-8.

## Key Points

• RYGB provides greater remission of metabolic comorbidities than SG.

• Cardiometabolic outcomes differ between RYGB and SG beyond weight loss.

• Benefits are more consistent for dyslipidemia and diabetes remission.

• Evidence derives exclusively from randomized controlled trials.

**Supplementary Information:**

The online version contains supplementary material available at 10.1007/s11695-026-08664-8.

## Introduction

Obesity is a chronic condition with a high worldwide prevalence and one of the main modifiable determinants of cardiovascular risk [1–4]. Excess visceral adiposity promotes a chronic low-grade inflammatory state associated with immunometabolic dysfunction, insulin resistance, and hormonal disturbances [5–8]. These mechanisms directly contribute to the development of type 2 diabetes mellitus (T2DM), systemic arterial hypertension, and dyslipidemia, which mediate much of the association between obesity and cardiovascular morbidity and mortality [5, 6]. Therefore, effective obesity control is essential to reduce cardiovascular events and improve long-term prognosis [3, 9, 10].

In this context, bariatric surgery—currently recognized as metabolic surgery—represents the most effective intervention for individuals with severe obesity [11–13]. Although conservative strategies are important initially, their long-term efficacy is limited [13, 14]. In contrast, surgical procedures promote substantial weight loss and anatomical and neurohormonal changes that lead to sustained improvements in glycemic control, lipid profile, and blood pressure [15–18]. Studies have demonstrated reductions in all-cause mortality, lower incidence of cardiovascular events, and higher remission rates of cardiometabolic comorbidities in patients undergoing surgery compared with those receiving medical therapy alone [16, 19–21].

Roux-en-Y gastric bypass (RYGB) has historically been used as a reference procedure because it combines gastric restriction with intestinal bypass and additional metabolic effects [16, 21, 22]. Sleeve gastrectomy (SG), currently the most frequently performed procedure worldwide, offers technical simplicity and comparable early outcomes [23–25]. The relative superiority of each technique over time, however, remains uncertain [12, 21, 24]. Evidence shows heterogeneous results regarding T2DM remission, lipid profile control, and reductions in inflammatory markers, particularly in long-term follow-up [26–28]. This inconsistency highlights a relevant scientific gap and justifies the need for rigorous quantitative syntheses comparing the two techniques across multiple temporal horizons.

Against this background, this systematic review and meta-analysis compares RYGB and SG regarding continuous metabolic markers—including lipid, glycemic, and blood pressure profiles, and C-reactive protein (CRP)—and remission of main cardiometabolic comorbidities (T2DM, hypertension, and dyslipidemia), stratified by short-term (1 year), mid-term (> 1 to < 10 years), and long-term (≥ 10 years) follow-up.

## Methods

### Protocol and Registration

This systematic review and meta-analysis was registered in the PROSPERO database (CRD420251266371) and conducted in accordance with PRISMA 2020 guidelines [29].

### Search Strategy and Study Selection

A systematic search was conducted in the electronic databases PubMed (MEDLINE), Embase, Web of Science, and the Cochrane Library, covering the period from database inception to December 17, 2025. The search strategy was developed based on the PICOTT framework and focused on terms related to cardiovascular risk after RYGB and SG (detailed search strategies are reported in the Appendix in the Supplement).

All records were imported into the Rayyan software [30]. Duplicates were removed automatically and reviewed manually. Two independent reviewers screened titles and abstracts, followed by a full-text assessment of eligible studies. Disagreements were resolved by a third reviewer.

### Eligibility Criteria

Inclusion in this meta-analysis was restricted to studies that met all of the following criteria: (1) randomized controlled trials (RCTs); (2) studies comparing RYGB and SG as primary (non-revisional) procedures, without concomitant use of obesity management medications; (3) studies that evaluated at least one of the following outcomes: glycated hemoglobin (HbA1c), fasting glucose, triglycerides, total cholesterol, high-density lipoprotein cholesterol (HDL), low-density lipoprotein cholesterol (LDL), blood pressure, CRP, dyslipidemia, hypertension, and T2DM; and (4) studies with a minimum follow-up of 1 year. Studies that did not meet at least one of these criteria were excluded.

### Data Extraction

After study selection, data were independently extracted by two reviewers, with discrepancies resolved by consensus and verification by a third author. Standardized Excel software was used to collect study design, setting, follow-up, sample size, demographic characteristics, body mass index (BMI), and baseline comorbidities (T2DM, dyslipidemia, and hypertension).

The continuous outcomes extracted were lipid profile parameters (total cholesterol, LDL cholesterol, HDL cholesterol, and triglycerides), fasting glucose, HbA1c, blood pressure levels, and CRP. Dichotomous outcomes included remission of T2DM, dyslipidemia, and hypertension. The definitions used for each comorbidity across the included studies are detailed in Supplementary Table S1.

### Risk of Bias and Certainty of Evidence

Risk of bias was independently assessed by two reviewers using the Cochrane RoB 2 tool [31], with disagreements resolved by a third reviewer. Given the number of correlated outcomes, GRADE was limited to binary outcomes to avoid redundancy and overestimation and conducted by a single reviewer [32].

### Statistical Analysis

Analyses were performed in R version 4.3.2 [33] using the meta and metafor packages, with statistical significance set at *p* < 0.05. For studies reporting continuous outcomes as medians, interquartile range (IQR), or range, means and standard deviations were estimated using the method of Wan et al., 2014 [34].

Continuous outcomes were analyzed as mean differences (MDs) and dichotomous outcomes as risk ratios (RRs), both with 95% confidence intervals (CIs), using the Inverse Variance and Mantel–Haenszel methods, respectively.

Heterogeneity was assessed using Cochran’s Q test (Chi²) and the I² statistic, with between-study variance estimated by tau-squared (τ²). Significant heterogeneity was defined as I² > 25% or *p* < 0.10 in the Q test guiding the use of random-effects models; fixed-effect models were applied otherwise. To test the stability of the findings, leave-one-out sensitivity analyses were conducted to assess robustness and explore sources of heterogeneity by sequential study exclusion.

## Results

### Study Selection and Eligibility

**Fig. 1:**
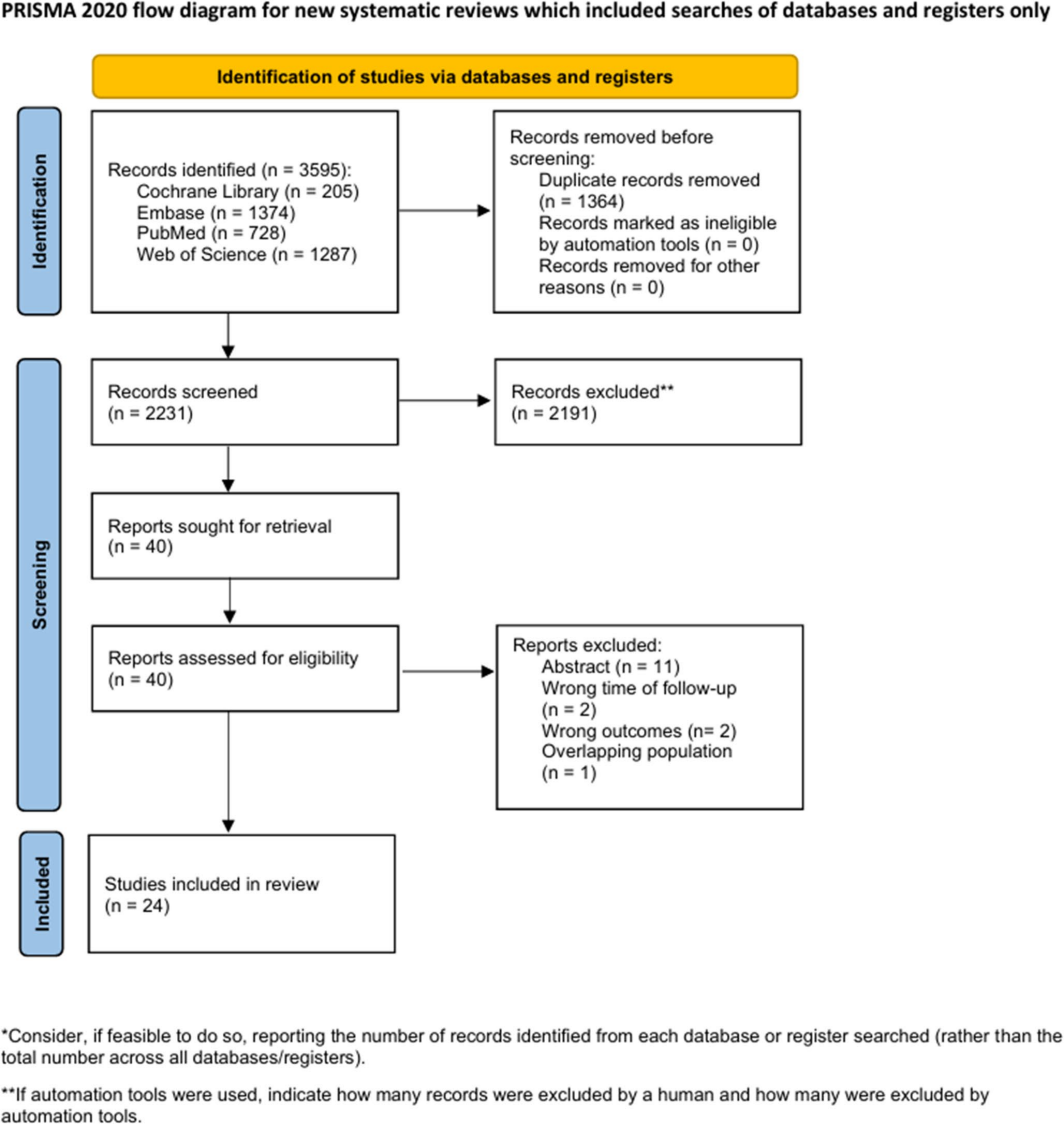
Study flow diagram

The systematic search initially identified 3,595 studies. After removing duplicates, 2,231 titles and abstracts were screened, and 40 articles were assessed in full text. Of these, 16 were excluded, mainly because they were conference abstracts or did not meet the predefined eligibility criteria. Consequently, 24 studies [11, 26, 27, 35–55] met al.l eligibility criteria and were included in the quantitative analysis (Fig. 1).

### Characteristics of Included Studies

The included studies consisted exclusively of randomized clinical trials published between 2011 and 2025, conducted across 33 centers in 14 countries, comprising a total of 2,890 participants. The overall mean age was 47.8 years (ranging from 35.2 to 67 years), with a predominance of female participants (71.42%). The mean BMI was 42 kg/m². The baseline prevalence of dyslipidemia, hypertension, and type 2 diabetes mellitus was comparable between the RYGB and SG groups (Table 1).

**Table 1:** Baseline characteristics of the included randomized controlled trials

Study	Follow-up (years)	Total *n* (RYGB : SG)	RYGB		SG		Mean age	BMI mean (kg/m²)		Dyslipidemia (*n*)		T2DM (*n*)		Hypertension (*n*)	
			Male	Female	Male	Female		RYGB	SG	RYGB	SG	RYGB	SG	RYGB	SG
Benaiges et al., 2024 [35]	1	36 (18 : 18)	02	16	03	15	52.12	43.5	43.2	18	18	05	06	09	11
Biter et al., 2024 [36]	5	240 (119 : 121)	39	80	34	87	48.5	46.4	45.5	65	71	62	54	87	83
By-Band- Sleeve et al., 2025 [37]	3	882 (462 : 420)	116	345	98	321	47.6	46.9	46.1	131	110	152	117	210	176
Casajoana et al., 2021 [38]	5	30 (15 : 15)	07	08	05	10	50.1	38.7	39.0	10	11	15	15	10	09
Ceperuelo- Mallafré et al., 2019 [39]	2	30 (15 : 15)	07	08	06	10	50.1	38.6	39.0	NR	NR	15	15	NR	NR
Grinlinton et al., 2025 [27]	10	114 (56 : 58)	23	33	26	32	46	42.2	41.9	29	35	56	58	38	36
Hofsø et al., 2019 [41]	1	109 (54 : 55)	14	40	22	32	47.7	42.4	42.1	21	28	54	55	37	36
Hauge et al., 2025 [40]	5	109 (54 : 55)	14	40	23	32	47.7	42.4	42.2	22	28	54	55	37	36
Kalinowski et al., 2016	1	72 (36 : 36)	13	23	10	26	44.4	48.6	46.1	31	31	14	12	30	25
Keidar et al., 2013 [43]	1	37 (19 : 18)	12	08	9	09	49.6	42.0	42.5	NR	NR	19	18	NR	NR
Lee et al., 2011 [45]	1	60 (30 : 30)	NR	NR	NR	NR	45	30.7	30.7	NR	NR	30	30	NR	NR
Murphy et al., 2017 [46]	1	114 (56 : 58)	23	33	26	32	46	42.2	41.9	29	35	56	58	38	36
Murphy et al., 2022 [47]	5	114 (56 : 58)	24	33	26	32	46	42.2	41.9	29	35	56	58	38	36
Pajecki et al., 2021 [48]	1	36 (18 : 18)	05	13	00	18	67	51.3	46.3	07	06	14	13	16	18
Pajecki et al., 2023 [49]	3	36 (18 : 18)	06	13	00	18	67	46.9	43.1	07	07	14	13	16	18
Salminen et al., 2018	5	628 (316 : 312)	56	260	58	254	43	43.3	43.7	45	39	49	52	103	111
Salminen et al., 2022 [26]	10	240 (119 : 121)	39	80	34	87	48.5	46.4	45.5	45	39	49	52	87	83
Peterli et al., 2018 [50]	5	217 (110 : 107)	31	79	30	77	42.6	44.2	43.6	53	68	28	26	64	64
Kraljevic et al., 2025 [44]	10	218 (110 : 107)	31	79	30	77	42.6	44.2	43.6	53	68	29	27	41	40
Schauer et al., 2012 [12]	1	100 (50 : 50)	21	29	11	39	48.2	37.0	36.0	44	40	50	50	35	30
Schauer et al., 2017 [11]	5	96 (49 : 47)	21	28	11	39	48.2	37.0	36.0	43	38	49	47	35	30
Tang et al., 2016 [53]	2	72 (38 : 34)	12	22	20	18	38.5	37.8	38.6	10	11	38	34	16	12
Vix et al., 2013 [54]	1	100 (45 : 55)	06	39	12	43	35.2	45.1	45.6	12	15	04	04	17	12
Yan et al., 2021 [55]	1	157 (77 : 80)	41	36	43	37	44	35.7	36.2	NR	NR	77	80	NR	NR

Abbreviations: n: Number; RYGB: Roux-en-Y gastric bypass surgery; SG: Sleeve gastrectomy; BMI: Body Mass Index; T2DM: Type 2 Diabetes Mellitus; NR: Not reported

### Quantitative Analysis

The main quantitative findings are summarized in Table 2, providing an integrated overview of the comparative effects of RYGB and SG across follow-up periods.

**Table 2:** Summary of findings

Outcomes		Follow-up analyses (years)	Studys (*n*)	Effect (IC95%)	I² (%)	*p*-value	Favours
Lipid profile	Total cholesterol (mmol/L)	1	8	MD -0.59 (-0.85 to -0.33)	72.6	< 0.001	RYGB
		> 1 < 10	7	MD -0.40 (-0.57 to -0.23)	56.4	< 0.001	RYGB
		≥ 10	2	MD -0,07 (-0,30 to 0,15)	0.0	0.516	NS
	LDL (mmol/L)	1	9	MD -0.55 (-0.79 to -30)	91.9	< 0.001	RYGB
		> 1 < 10	8	MD -0.35 (-0.51 to -0.23)	66.7	< 0.001	RYGB
		≥ 10	2	MD -0.14 (-0.30 to 0.02)	0.0	0.079	NS
	HDL (mmol/L)	1	8	MD -0.07 (-0.16 to 0.03)	86.0	0.157	NS
		> 1 < 10	7	MD -0.07 (0.02 to 0.12)	54.5	0.010	RYGB
	Triglyceridis (mmol/L)	1	8	MD -0.10 (-0.24 to 0.05)	85.9	0.185	NS
		> 1 < 10	7	MD -0.22 (-0.43 to -0.02)	91.9	0.033	RYGB
Glycemic	HbA1c (%)	1	10	MD -0.30 (-0.57 to -0.02)	74.5	0.036	RYGB
		> 1 < 10	8	MD -0.13 (-0.29 to 0.02)	44.2	0.091	NS
		≥ 10	2	MD 0.02 (-0.20 to 0.25)	0.0	0.851	NS
	Fasting glucose (mmol/L)	1	8	MD -0.26 (-0.56 to 0.04)	75.5	0.094	NS
		> 1 < 10	7	MD -0.32 (-0.87 to 0.24)	61.2	0.260	NS
		≥ 10	2	MD 0.11 (-0.28 to 0.51)	31.1	0.566	NS
Pressure	SBP (mmHg)	1	4	MD -0.59 (-4.49 to 3.31)	0.0	0.767	NS
		> 1 < 10	3	MD -0.16 (-3.78 to 3.47)	0.0	0.933	NS
	DBP (mmHg)	1	4	MD -1.67 (-3.68 to 0.33)	0.0	0.102	NS
		> 1 < 10	3	MD -1.46 (-4.88 to 1.96)	49.2	0.402	NS
IM	CRP (mg/L)	1	2	MD -0.54 (-0.90 to 0.18)	0.0	0.003	RYGB
Comorbidity remission	Dyslipidemia	1	4	RR 1.68 (1.05 to 2.69)	66.7	0.032	RYGB
		> 1 < 10	9	RR 1.41 (1.26 to 1.57)	11.9	< 0.001	RYGB
		≥ 10	2	RR 1.04 (0.36 to 3.04)	62.4	0.938	NS
	Hypertension	1	3	RR 1.04 (0.83 to 1.31)	0.0	0.713	NS
		> 1 < 10	7	RR 1.00 (0.71 to 1.42)	69.1	0.978	NS
		≥ 10	2	RR 1.34 (0.90 to 1.98)	79.9	0.148	NS
	T2DM (HbA1c < 6%)	1	7	RR 1.40 (1.16 to 1.69)	0.0	< 0.001	RYGB
		> 1 < 10	6	RR 1.20 (0.89 to 1.62)	52.8	0.234	NS
		≥ 10	3	RR 1.30 (0.91 to 1.86)	0.0	0.151	NS
	T2DM (HbA1c < 6,5%)	1	3	RR 1.34 (0.91 to 1.97)	74.4	0.020	NS
		> 1 < 10	6	RR 1.24 (0.95 to 1.63)	61.5	0.118	NS

Abbreviations: n: Number; CI: Confidence Interval; RYGB: Roux-en-Y gastric bypass surgery; SG: Sleeve gastrectomy; NS: Not significant; MD: Mean Difference; RR: Risk Ratio; LDL: Low-density lipoprotein cholesterol; HDL: High-density lipoprotein cholesterol; HbA1c: Glycated hemoglobin; SBP: Sistolic blood pressure; DBP: Diastolic blood pressure; IM: Inflammatory markers; CRP: C-reactive protein; T2DM: Diabetes Mellitus type 2

#### Lipid Outcomes

In the analysis of total cholesterol, a greater reduction was observed after RYGB compared with SG at 1 year (MD = − 0.59; 95% CI, − 0.85 to − 0.33; *p* < 0.001; I² = 72.6%), a finding that persisted in the pooled analysis from 1 to 10 years (MD = − 0.40; 95% CI, − 0.57 to − 0.23; *p* < 0.001; I² = 56.4%). At 10 years, no statistically significant difference was observed (MD = − 0.07; 95% CI, − 0.30 to 0.15; *p* = 0.516; I² = 0.0%) (Figure S1).

LDL cholesterol showed a similar pattern, with greater reductions favoring RYGB at 1 year (MD = − 0.55; 95% CI, − 0.79 to − 0.30; *p* < 0.001; I² = 91.9%) and between 1 and 10 years (MD = − 0.37; 95% CI, − 0.51 to − 0.23; *p* < 0.001; I² = 66.7%). At 10 years, the difference was not statistically significant (MD = − 0.14; 95% CI, − 0.30 to 0.02; *p* = 0.079; I² = 0.0%), although the point estimate favored RYGB was observed (Figure S2).

The analysis of triglycerides did not demonstrate statistical significance at 1 year (MD = − 0.10; 95% CI, − 0.24 to 0.05; *p* = 0.185; I² = 85.9%), but showed superiority of RYGB in the 1–10 year interval (MD = − 0.22; 95% CI, − 0.43 to − 0.02; *p* = 0.033; I² = 91.9%) (Figure S3).

For HDL cholesterol, no significant difference was observed at 1 year (MD = − 0.07; 95% CI, − 0.16 to 0.03; *p* = 0.157; I² = 86.0%). However, in the combined analysis from 1 to 10 years, RYGB was associated with higher HDL levels (MD = 0.07; 95% CI, 0.02 to 0.12; *p* = 0.010; I² = 54.5%) (Figure S4).

#### Glycemic Outcomes

HbA1c showed a greater reduction after RYGB at 1 year (MD = − 0.30%; 95% CI, − 0.57 to − 0.02; *p* = 0.036; I² = 74.5%). However, no significant differences were observed in the pooled analysis from 1 to 10 years (MD = − 0.13; 95% CI, − 0.29 to 0.02; *p* = 0.091; I² = 44.2%) or in the isolated 10-year analysis (MD = 0.02; 95% CI, − 0.20 to 0.25; *p* = 0.851; I² = 0.0%) (Figure S5).

For fasting glucose, a tendency toward greater reduction with RYGB was observed at 1 year (MD = − 0.26; 95% CI, − 0.56 to 0.04; *p* = 0.094; I² = 75.5%), in the combined 1–10 year analysis (MD = − 0.32; 95% CI, − 0.87 to 0.24; *p* = 0.260; I² = 61.2%), and at 10 years (MD = 0.11; 95% CI, − 0.28 to 0.51; *p* = 0.566; I² = 31.1%). Nevertheless, none of these differences reached statistical significance (Figure S6).

#### Blood Pressure Outcomes

Systolic blood pressure did not differ significantly between groups at 1 year (MD = − 0.59; 95% CI, − 4.49 to 3.31; *p* = 0.767; I² = 0.0%) or in the pooled 10-year analysis (MD = − 0.16; 95% CI, − 3.78 to 3.47; *p* = 0.933; I² = 0.0%) (Figure S7).

Similarly, diastolic blood pressure showed no significant differences between RYGB and SG at 1 year (MD = − 1.67; 95% CI, − 3.68 to 0.33; *p* = 0.102; I² = 0.0%) or in the combined 1–10 year analysis (MD = − 1.46; 95% CI, − 4.88 to 1.96; *p* = 0.402; I² = 49.2%) (Figure S8).

#### Inflammatory Markers

CRP showed a significantly greater reduction in the RYGB group at 1 year (MD = − 0.54; 95% CI, − 0.90 to − 0.18; *p* = 0.003; I² = 0.0%) (Figure S9).

#### Remission of Medical Comorbidities

Remission of dyslipidemia favored RYGB at 1 year (RR = 1.68; 95% CI, 1.05 to 2.69; *p* = 0.032; I² = 66.7%) and in the pooled 1–10 year analysis (RR = 1.41; 95% CI, 1.26 to 1.57; *p* < 0.001; I² = 11.9%). At 10 years, however, no significant difference was observed between procedures (RR = 1.04; 95% CI, 0.36 to 3.04; *p* = 0.151; I² = 62.4%) (Figure S10).

For remission of hypertension, no significant differences were identified at any time point: 1 year (RR = 1.04; 95% CI, 0.83 to 1.31; *p* = 0.713; I² = 0.0%), > 1 < 10 years (RR = 1.00; 95% CI, 0.71 to 1.42; *p* = 0.978; I² = 69.1%), or 10 years (RR = 1.34; 95% CI, 0.90 to 1.98; *p* = 0.148; I² = 79.9%) (Figure S11).

Regarding T2DM, remission outcomes were stratified according to the HbA1c threshold used to define remission (HbA1c < 6.0% or HbA1c < 6.5%). For the HbA1c < 6.0% subgroup, higher remission rates with RYGB were observed at 1 year (RR = 1.40; 95% CI, 1.16 to 1.69; *p* < 0.001; I² = 0.0%). However, no significant differences were found between 1 and 10 years (RR = 1.20; 95% CI, 0.89 to 1.62; *p* = 0.234; I² = 52.8%) or at 10 years (RR = 1.30; 95% CI, 0.91 to 1.86; *p* = 0.151; I² = 0.0%).

For the HbA1c < 6.5% subgroup, no significant differences were observed at 1 year (RR = 1.34; 95% CI, 0.91 to 1.97; *p* = 0.140; I² = 74.4%) or between 1 and 10 years (RR = 1.24; 95% CI, 0.95 to 1.63; *p* = 0.118; I² = 61.5%) (Figure S12).

### Sensitivity Analysis

In the leave-one-out analysis for HDL cholesterol at 1 year, exclusion of Lee et al., 2011 [45] resulted in a statistically significant effect favoring RYGB, whereas exclusion of Pajecki et al., 2023 [49] substantially reduced heterogeneity. In the pooled 1–10 years analysis, exclusion of Peterli et al., 2018 [50] markedly reduced heterogeneity (Figure S3).

Regarding triglycerides at 12 months, exclusion of Vix et al., 2013 [54] yielded a significant effect favoring RYGB, while removal of Pajecki et al., 2023 [49] substantially reduced heterogeneity. Similarly, in the pooled 1–10 year analysis, omission of Schauer et al., 2017 [11] favored RYGB, and exclusion of Pajecki et al., 2023 [49] again reduced heterogeneity (Figure S4).

For HbA1c at 1 year, omission of Lee et al., 2011 [45] substantially reduced heterogeneity, conversely, exclusion of Ceperuelo-Mallafré et al., 2019 [39], Hofsø et al., 2019 [41], Pajecki et al., 2023 [49], or Schauer et al., 2012 [52] eliminated the statistical significance. In the 1–10 years analysis, heterogeneity was more evenly distributed, with Schauer et al., 2017 [11], Peterli et al., 2018 [50], and Salminen et al., 2022 [26] contributing most to I² reduction. Additionally, exclusion of By-Band-Sleeve et al., 2025 [37] or Tang et al., 2016 [53] resulted in a statistically significant effect favoring RYGB (Figure S5).

For diastolic blood pressure between 1 and 10 years, removal of Schauer et al., 2017 [11] favored RYGB, whereas exclusion of Lee et al., 2011 [45] modestly reduced heterogeneity (Figure S8).

In the analysis of dyslipidemia remission at 1 year, exclusion of Murphy et al., 2017 [46] favored RYGB, while the remaining studies contributed relatively homogeneously to the observed heterogeneity (Figure S10). Finally, for hypertension remission in the 1–10 years analysis, omission of Hauge et al., 2025 [40] resulted in a marked reduction in heterogeneity and yielded a borderline effect favoring RYGB (Figure S11).

In the HbA1c < 6.5% subgroup between 1 and 10 years, leave-one-out sensitivity analysis showed that exclusion of the study by Tang et al., 2016 [53] reduced heterogeneity to 0% and favored T2DM remission in the RYGB group (Figure S13).

### Risk of Bias and GRADE Assessment

**Fig. 2:**
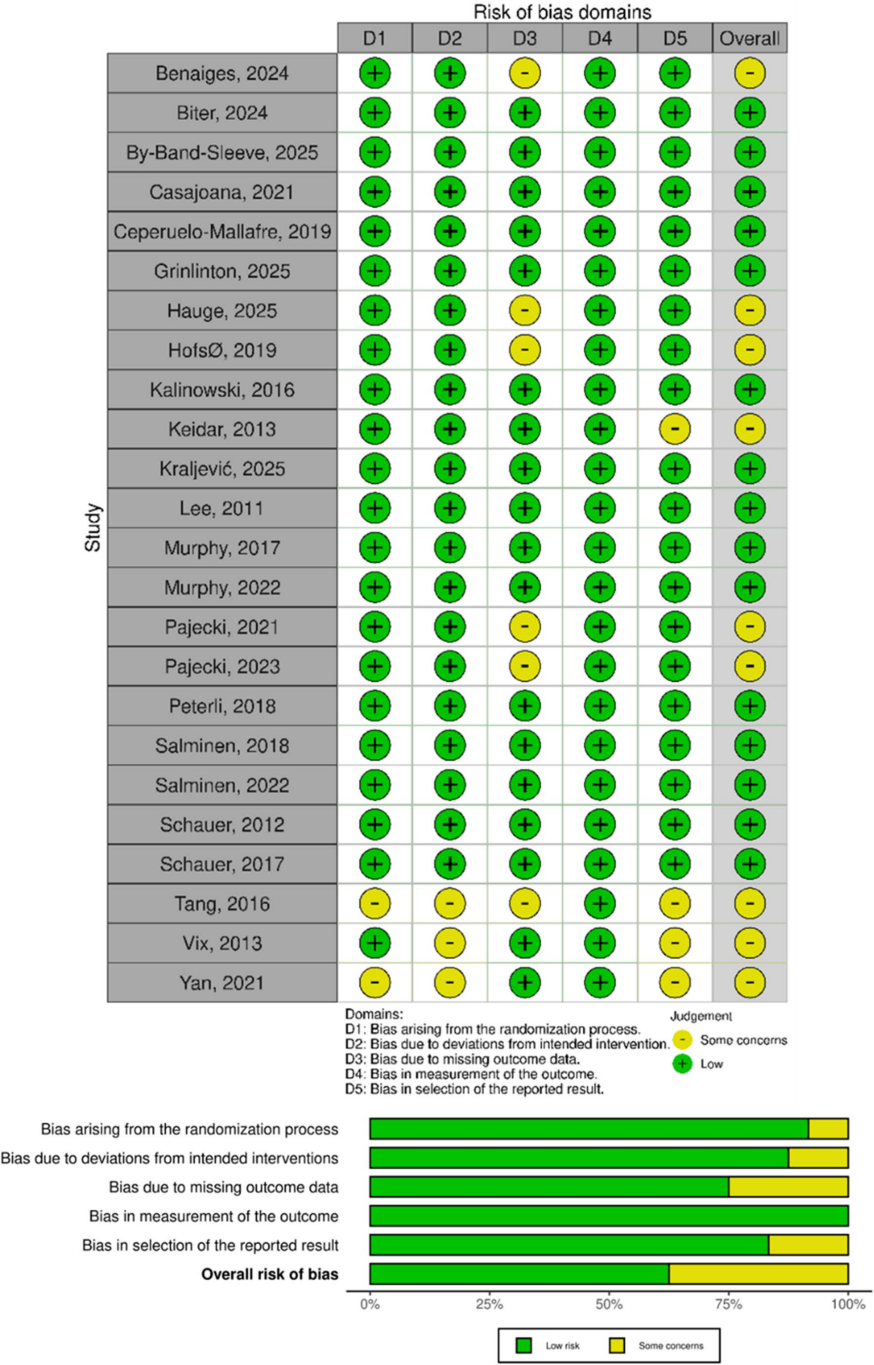
Risk of bias assessment using RoB 2

Fifteen studies were rated as low risk of bias, and nine were rated as “some concerns” (Fig. 2). According to GRADE, risk of bias and indirectness were considered “not serious” for all dichotomous outcomes. Inconsistency and imprecision were noted across outcomes, ranging from not serious to very serious, resulting in overall certainty of evidence ranging from very low to high (Table S2).

## Discussion

Evaluating cardiometabolic outcomes is critical, as obesity is strongly associated with increased arterial stiffness and heightened cardiovascular risk [56]. The findings of this meta-analysis of randomized clinical trials reveal a consistent temporal differentiation between surgical techniques. Roux-en-Y gastric bypass confers superior cardiometabolic benefits compared with sleeve gastrectomy in the short term, particularly during the first postoperative year. Nevertheless, this advantage attenuates overtime, suggesting long-term convergence in most metabolic outcomes, although evidence remains limited.

With respect to lipid outcomes, RYGB demonstrated sustained superiority in dyslipidemia remission, accompanied by greater reductions in total cholesterol and low-density lipoprotein (LDL) cholesterol at both 1 year and during mid-term follow-up (> 1 to < 10 years). These findings align with prior studies reporting more pronounced lipid improvements after RYGB compared with SG [57–59]. For triglycerides, a delayed benefit favoring RYGB emerged in the pooled mid-term analysis. This temporal pattern contrasts with observations by Szczuko et al. [59], who described an early and abrupt triglyceride decline following RYGB, whereas SG was associated with a more gradual trajectory. Despite this, the mid-term advantage of RYGB observed in the present analysis is consistent with the broader literature [58–60].

Concerning high-density lipoprotein (HDL) cholesterol, obesity is characterized not only by reduced circulating levels but also by impaired functional properties of HDL particles [61–64]. Within this framework, RYGB was associated with more favorable quantitative HDL changes between 1 and 10 years, potentially mediated by bile acid–driven remodeling of lipid transport pathways [64]. Nonetheless, emerging evidence suggests a dissociation between HDL quantity and function. Several studies indicate that SG may preserve cholesterol efflux capacity, particularly via the ABCA1-dependent pathway, which may be compromised after bypass surgery [65, 66]. Despite these mechanistic differences, both procedures appear to restore vascular protection over the long term, likely through shared effects on insulin sensitivity and reductions in visceral adiposity [67].

In the glycemic domain, RYGB demonstrated early superiority, with greater reductions in glycated hemoglobin at 1 year and between 1 and 10 years and higher remission rates of T2DM depending on the remission criterion applied. These findings suggest that the apparent superiority of RYGB may vary according to the remission threshold and the influence of individual studies. Nevertheless, the early glycemic advantage is consistent with prior meta-analyses reporting a substantially higher likelihood of strict glycemic remission (HbA1c < 6.0%) following RYGB, attributed to enhanced incretin signaling and rapid improvements in insulin sensitivity induced by intestinal rearrangement [68]. Fasting glucose levels, by contrast, did not differ significantly between procedures at any time point, suggesting comparable suppression of hepatic glucose production with both techniques [69].

Over longer follow-up (> 1 to < 10 years), the initial glycemic advantage of RYGB diminished, with convergence of HbA1c levels [60, 70]. This pattern suggests that long-term glycemic control is more strongly influenced by residual β-cell functional reserve and patient-specific metabolic characteristics than by the choice of surgical technique alone [54, 71].

For hypertension, consistent equivalence between RYGB and SG was observed across all time horizons. Prior studies similarly indicate that SG achieves the metabolic threshold required for blood pressure remission in most patients, yielding long-term outcomes comparable to those of RYGB [72, 73]. Notably, an exception has been described among individuals with BMI > 50 kg/m², in whom RYGB appears to confer superior blood pressure control, suggesting that the additional metabolic potency of bypass may be clinically relevant in settings of severe metabolic resistance [74].

Systemic inflammation represents another key mechanistic link between obesity and cardiovascular disease. CRP, a widely used marker of low-grade inflammation, declined after both procedures; however, RYGB was associated with a significantly greater reduction at 1 year. This finding suggests more pronounced early inflammatory modulation following bypass, potentially mediated by enhanced regulation of the NLRP3 inflammasome and downstream proinflammatory cytokines [75].

Despite the exclusive inclusion of randomized clinical trials, this study has important limitations. Substantial statistical heterogeneity was observed for several outcomes, likely reflecting differences in study populations, surgical techniques, perioperative protocols, and definitions of remission. Sensitivity analyses identified specific trials (Pajecki et al., 2023 [50]; Schauer et al., 2017 [11]; Peterli et al., 2018 [51]; Lee et al., 2011 [46]) as major contributors to this variability. Additionally, attrition over extended follow-up in some trials may introduce selection bias and overestimate long-term benefits. Only three trials provided data beyond 10 years, limiting the precision and generalizability of very long-term estimates.

In addition, most studies did not report standardized management of cardiometabolic medications. Even though discontinuation of these therapies may represent a beneficial clinical outcome after bariatric surgery, the lack of standardized reporting could have influenced some metabolic outcomes. Furthermore, the limited data on major adverse cardiovascular events and all-cause mortality precludes definitive conclusions regarding clinical relevance. Finally, weight loss—an important mediator of cardiometabolic outcomes—was not analyzed in this study, as it has been well-documented in prior literature and fell outside the primary scope of this analysis; however, its absence limits the ability to determine whether the observed effects are driven by differences in weight reduction.

## Conclusion

RYGB is associated with greater short- and mid-term cardiometabolic benefits compared with SG; however, these differences appear to attenuate over time. Interpretation of ≥ 10-year outcomes should be cautious, as the evidence is limited. Larger studies with longer follow-up are needed to confirm these findings.

## Supplementary Information

Below is the link to the electronic supplementary material.

Supplementary Material 1

## Data Availability

No datasets were generated or analyzed during thecurrent study.

## Abbreviations

BMI: Body mass index
CI: Confidence Interval
CRP: C-reactive protein
GRADE: Grading of Recommendations Assessment, Development and Evaluation
HbA1c: Glycated hemoglobin
HDL: High-density lipoprotein cholesterol
HR: Hazard ratio
LDL: Low-density lipoprotein cholesterol
MD: Mean difference
MH: Mantel–Haenszel method
PRISMA: Preferred Reporting Items for Systematic Reviews and Meta-Analyses
RCT: Randomized controlled trial
RoB 2: Risk of Bias 2
RR: Risk ratio
RYGB: Roux-en-Y gastric by-pass
SG: Sleeve gastrectomy
SBP: Systolic blood pressure
T2DM: Type 2 diabetes mellitus
DBP: Diastolic blood pressure
VI: Inverse variance method
