# Supplementary material for: Roux-en-Y Gastric Bypass versus Sleeve Gastrectomy for Cardiometabolic Outcomes: A Systematic Review and Meta-analysis of Randomized Controlled Trials
Source: Obes Surg. 2026 Apr 29;36(6):3247–58. doi: 10.1007/s11695-026-08664-8 (PMC13249783; doi:10.1007/s11695-026-08664-8)
Supplement: Supplementary file 1 — Supplementary Material 1 [file 11695_2026_8664_MOESM1_ESM.pdf]

Supplementary Online Content

Appendix. Search strategies.

Table S1. Definitions of comorbidity remission used in the included studies.

Figure S1. Meta-analysis of mean differences in total cholesterol between RYGB and SG, including leave-one-out sensitivity analyses.

Figure S2. Meta-analysis of mean differences in low-density lipoprotein cholesterol between RYGB and SG, including leave-one-out sensitivity analyses.

Figure S3. Meta-analysis of mean differences in high-density lipoprotein cholesterol between RYGB and SG, including leave-one-out sensitivity analyses.

Figure S4. Meta-analysis of mean differences in triglycerides between RYGB and SG, including leave-one-out sensitivity analyses.

Figure S5. Meta-analysis of mean differences in glycated hemoglobin between RYGB and SG, including leave-one-out sensitivity analyses.

Figure S6. Meta-analysis of mean differences in fasting glucose between RYGB and SG, including leave-one-out sensitivity analyses.

Figure S7. Meta-analysis of mean differences in systolic blood pressure between RYGB and SG, including leave-one-out sensitivity analyses.

Figure S8. Meta-analysis of mean differences in diastolic blood pressure between RYGB and SG, including leave-one-out sensitivity analyses.

Figure S9. Meta-analysis of mean differences in C-reactive protein between RYGB and SG, including leave-one-out sensitivity analyses.

Figure S10. Meta-analysis of risk ratios for dyslipidemia remission comparing RYGB and SG, including leave-one-out sensitivity analyses.

Figure S11. Meta-analysis of risk ratios for hypertension remission comparing RYGB and SG, including leave-one-out sensitivity analyses.

Figure S12. Meta-analysis of risk ratios for type 2 diabetes remission defined as HbA1c < 6%, comparing RYGB and SG, including leave-one-out sensitivity analyses.

Figure S13. Meta-analysis of risk ratios for type 2 diabetes remission defined as HbA1c < 6.5%, comparing RYGB and SG, including leave-one-out sensitivity analyses.

Table S2. GRADE evidence profile for clinically important binary outcomes.

References

This supplementary material has been provided by the authors to give readers additional information about their work.

© 2016 American Medical Association. All rights reserved.

Appendix: Search strategies

Search for Medline, Cochrane Library and Web of Science

("Roux-en-Y gastric bypass" OR "RYGB" OR "Roux-Y" OR "Roux-en-Y" OR "gastric bypass")
AND
("Sleeve Gastrectomy" OR "sleeve gastrectomy" OR "SG" OR "vertical gastrectomy" OR "Longitudinal Gastrectomy" OR "Longitudinal Gastric Sleeve" OR "Vertical Sleeve Gastrectomy")
AND
("HbA1c" OR "Glycated Hemoglobin" OR "Triglycerides" OR "Total cholesterol" OR "Cholesterol" OR "High-density lipoprotein" OR "HDL" OR "Low-density lipoprotein" OR "LDL" OR "Blood Pressure" OR "C-reactive protein" OR "Dyslipidemia" OR "lipid metabolism disorder" OR "Blood measurements" OR "blood sampling" OR "Biochemical Outcomes" OR "metabolic outcomes")

Search for Embase

("Roux-en-Y gastric bypass":ti,ab OR "RYGB":ti,ab OR "Roux-Y":ti,ab OR "Roux-en-Y":ti,ab OR "gastric bypass":ti,ab)
AND
("Sleeve Gastrectomy":ti,ab OR "sleeve gastrectomy":ti,ab OR "SG":ti,ab OR "vertical gastrectomy":ti,ab OR "Longitudinal Gastrectomy":ti,ab OR "Longitudinal Gastric Sleeve":ti,ab OR "Vertical Sleeve Gastrectomy":ti,ab)
AND
("HbA1c":ti,ab OR "Glycated Hemoglobin":ti,ab OR "Triglycerides":ti,ab OR "Total cholesterol":ti,ab OR "Cholesterol":ti,ab OR "Remnant Cholesterol":ti,ab OR "High-density lipoprotein":ti,ab OR "HDL":ti,ab OR "Low-density lipoprotein":ti,ab OR "LDL":ti,ab OR "Blood Pressure":ti,ab OR "C-reactive protein":ti,ab OR "Dyslipidemia":ti,ab OR "lipid metabolism disorder":ti,ab OR "remnant cholesterol":ti,ab OR "Blood measurements":ti,ab OR "blood sampling":ti,ab OR "Biochemical Outcomes":ti,ab OR "metabolic outcomes":ti,ab)

© 2016 American Medical Association. All rights reserved.

Table S1. Definitions of comorbidity remission used in the included studies.

Study	Definition of T2DM remission
Benaiges et al., 2024	HbA1c < 6% + fasting glucose < 5,6 mmol/L
Casajoana et al., 2021	
Ceperuelo-Mallafré et al., 2019	
Grinlinton et al., 2025	
Kalinowski et al., 2016	
Kraljevic et al., 2025	
Murphy et al., 2017	
Murphy et al., 2022	
Peterli et al., 2018	
Salminen et al., 2018	
Tang et al., 2016	
Biter et al., 2024	HbA1c < 6% + fasting glucose < 7 mmol/L
Hauge et al., 2025	HbA1c < 6%
Hofsø et al., 2019	
Pajecki et al., 2021	
Schauer et al., 2012	
Schauer et al., 2017	
By-Band-Sleeve et al., 2025	HbA1c < 6.5%
Lee et al., 2011	
Salminen et al., 2022	

Study	Definition of Parcial T2DM remission
Hauge et al., 2025	HbA1c < 6.5%
Murphy et al., 2022	HbA1c < 6.5%
Schauer et al., 2017	
Tang et al., 2016	

Study	Definition of dyslepdemia remission
Benaiges et al., 2024	LDL < 3 mmol/L + No medication
Salminen et al., 2018	
Salminen et al., 2022	
Biter et al., 2024	Total cholesterol ≤ 5mmol + No medication
Casajoana et al., 2021	No medication
Hauge et al., 2025	
Hofsø et al., 2019	
Kraljevic et al., 2025	
Murphy et al., 2017	
Murphy et al., 2022	
Schauer et al., 2012	
Schauer et al., 2017	
Peterli et al., 2018	
Tang et al., 2016	

Study	Definition of hypertension remission
Biter et al., 2024	SBP < 120 mmHg and DBP < 80 mmHg + No medication
By-Band- Sleeve et al., 2025	SBP < 130 mmHg and DBP < 85 mmHg + No medication
Benaiges et al., 2024	No medication
Casajoana et al., 2021	
Hauge et al., 2025	
Kraljevic et al., 2025	
Murphy et al., 2017	
Salminen et al., 2018	
Salminen et al., 2022	
Schauer et al., 2012	
Peterli et al., 2018	
Tang et al., 2016	

Abbreviations: HbA1c: glycated hemoglobin; LDL: low-density lipoprotein cholesterol; SBP: systolic blood pressure; DBP: diastolic blood pressure.

© 2016 American Medical Association. All rights reserved.

Figure S1. Meta-analysis of mean differences in total cholesterol between RYGB and SG, including leave-one-out sensitivity analyses.

A. 1 year

Study	RYGB Mean	RYGB SD	RYGB Total	SG Mean	SG SD	SG Total	Weight	Mean Difference IV, Random, 95% CI
Benaiges, 2024	4.98	0.96	15	5.63	0.94	15	8.3%	-0.65 [-1.33; 0.03]
Ceperuelo-Mallafré, 2019	3.80	1.05	15	4.80	0.97	15	7.7%	-1.00 [-1.72; -0.28]
Hofsø, 2019	3.80	0.73	54	4.60	0.74	55	15.6%	-0.80 [-1.08; -0.52]
Lee, 2011	4.20	0.69	30	5.38	1.74	30	8.4%	-1.18 [-1.85; -0.51]
Salminen, 2022	4.30	1.03	105	4.70	0.53	110	16.7%	-0.40 [-0.62; -0.18]
Schauer, 2012	4.50	0.91	50	4.87	0.96	49	13.7%	-0.37 [-0.74; -0.00]
Vix, 2013	4.41	0.57	45	5.21	1.13	55	14.3%	-0.80 [-1.14; -0.46]
Yan, 2021	4.60	1.00	77	4.60	0.90	80	15.2%	0.00 [-0.30; 0.30]
**Total (95% CI)**			**391**			**409**	**100.0%**	**-0.59 [-0.85; -0.33]**

Heterogeneity: $Tau^2 = 0.0941$; $Chi^2 = 25.54$, df = 7 (P = 0.0006); $I^2 = 72.6\%$
Test for overall effect: Z = -4.41 (P < 0.001)

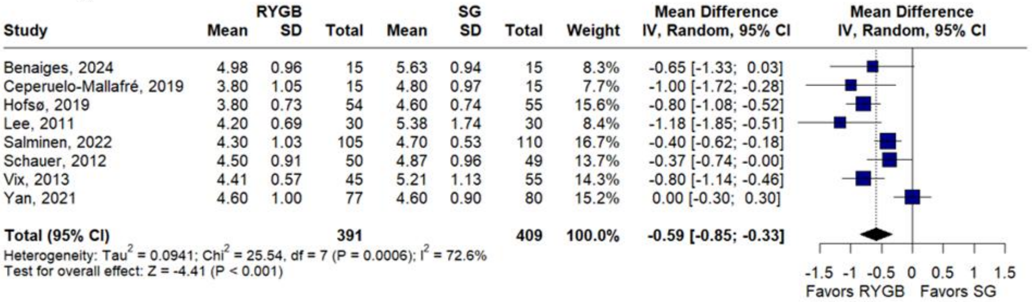

Study	MD	95%-CI	I2
Omitting Benaiges, 2024	-0.59	[-0.88; -0.30]	76.3%
Omitting Ceperuelo-Mallafré, 2019	-0.55	[-0.83; -0.28]	74.7%
Omitting Hofsø, 2019	-0.55	[-0.85; -0.26]	70.4%
Omitting Lee, 2011	-0.53	[-0.79; -0.27]	72.2%
Omitting Salminen, 2022	-0.63	[-0.94; -0.32]	75.2%
Omitting Schauer, 2012	-0.63	[-0.93; -0.33]	75.9%
Omitting Vix, 2013	-0.56	[-0.85; -0.26]	73.2%
Omitting Yan, 2021	-0.67	[-0.88; -0.45]	50.1%
**Random effects model**	**-0.59**	**[-0.85; -0.33]**	**72.6%**

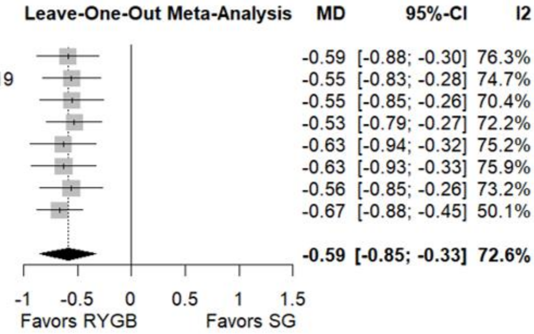

B. Between 1 and 10 years

Study	RYGB Mean	RYGB SD	RYGB Total	SG Mean	SG SD	SG Total	Weight	Mean Difference IV, Random, 95% CI
By-Band-Sleeve, 2025	4.40	1.00	461	5.00	1.00	419	25.8%	-0.60 [-0.73; -0.47]
Casajoana, 2021	4.09	0.70	14	4.74	0.90	14	6.5%	-0.65 [-1.25; -0.05]
Hauge, 2025	4.10	1.10	54	4.60	1.11	55	10.9%	-0.50 [-0.91; -0.09]
Murphy, 2022	5.10	1.00	50	5.10	1.00	53	12.0%	0.00 [-0.39; 0.39]
Peterli, 2018	4.83	0.82	53	5.07	1.11	68	13.7%	-0.24 [-0.58; 0.10]
Salminen, 2022	4.60	0.96	91	4.90	0.50	97	20.2%	-0.30 [-0.52; -0.08]
Tang, 2016	4.20	1.00	38	4.70	0.80	34	10.9%	-0.50 [-0.92; -0.08]
**Total (95% CI)**			**761**			**740**	**100.0%**	**-0.40 [-0.57; -0.23]**

Heterogeneity: $Tau^2 = 0.0250$; $Chi^2 = 13.77$, df = 6 (P = 0.0323); $I^2 = 56.4\%$
Test for overall effect: Z = -4.58 (P < 0.001)

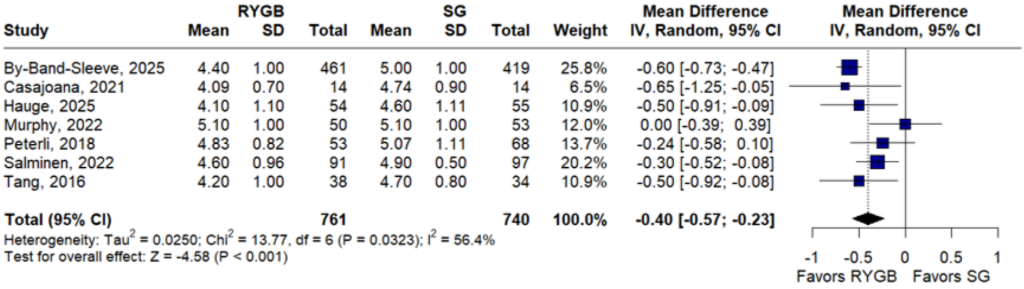

Study	MD	95%-CI	I2
Omitting By-Band-Sleeve, 2025	-0.32	[-0.46; -0.17]	8.8%
Omitting Casajoana, 2021	-0.38	[-0.56; -0.20]	62.7%
Omitting Hauge, 2025	-0.38	[-0.58; -0.19]	63.6%
Omitting Murphy, 2022	-0.46	[-0.61; -0.30]	35.7%
Omitting Salminen, 2022	-0.42	[-0.62; -0.22]	54.7%
Omitting Peterli, 2018	-0.42	[-0.61; -0.23]	58.2%
Omitting Tang, 2016	-0.38	[-0.58; -0.19]	63.6%
**Random effects model**	**-0.40**	**[-0.57; -0.23]**	**56.4%**

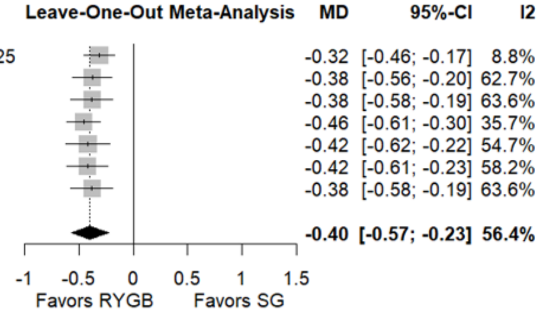

C. 10 years

Study	RYGB Mean	RYGB SD	RYGB Total	SG Mean	SG SD	SG Total	Weight	Mean Difference IV, Fixed, 95% CI
Kraljević, 2025	4.70	0.90	58	4.90	1.20	69	36.8%	-0.20 [-0.57; 0.17]
Salminen, 2022	4.50	0.93	86	4.50	0.99	96	63.2%	0.00 [-0.28; 0.28]
**Total (95% CI)**			**144**			**165**	**100.0%**	**-0.07 [-0.30; 0.15]**

Heterogeneity: $Tau^2 = 0$; $Chi^2 = 0.73$, df = 1 (P = 0.3942); $I^2 = 0.0\%$
Test for overall effect: Z = -0.65 (P = 0.516)

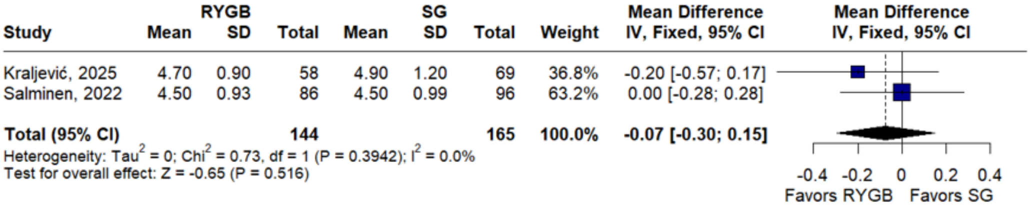

Abbreviations: RYGB: Roux-en-Y gastric bypass surgery; SG: Sleeve gastrectomy; CI: Confidence Interval; SD: Standard Deviation; IV: Inverse Variance; MD: Mean Difference.

© 2016 American Medical Association. All rights reserved.

Figure S2. Meta-analysis of mean differences in low-density lipoprotein cholesterol between RYGB and SG, including leave-one-out sensitivity analyses.

A. 1 year

Study	RYGB Mean	RYGB SD	RYGB Total	SG Mean	SG SD	SG Total	Weight	Mean Difference IV, Random, 95% CI
Benaiges, 2024	3.02	0.50	15	3.71	0.84	15	8.9%	-0.69 [-1.18; -0.20]
Ceperuelo-Mallafré, 2019	2.03	1.01	15	2.86	0.84	15	6.9%	-0.83 [-1.49; -0.17]
Hofsø, 2019	2.12	0.77	54	2.80	0.78	55	11.7%	-0.68 [-0.97; -0.39]
Lee, 2011	2.51	0.56	30	3.54	1.06	30	9.8%	-1.03 [-1.46; -0.60]
Pajecki, 2023	2.16	0.20	18	2.67	0.24	18	13.3%	-0.51 [-0.65; -0.37]
Salminen, 2022	2.30	0.51	104	2.60	1.05	109	12.6%	-0.30 [-0.52; -0.08]
Schauer, 2012	2.43	0.70	50	2.85	0.80	49	11.6%	-0.42 [-0.72; -0.12]
Vix, 2013	2.48	0.61	45	3.40	0.90	55	11.6%	-0.92 [-1.22; -0.62]
Yan, 2021	2.80	0.30	77	2.70	0.40	80	13.6%	0.10 [-0.01; 0.21]
**Total (95% CI)**			**408**			**426**	**100.0%**	**-0.55 [-0.79; -0.30]**

Heterogeneity: $Tau^2 = 0.1118$; $Chi^2 = 98.28$, df = 8 ($P < 0.0001$); $I^2 = 91.9\%$
Test for overall effect: $Z = -4.37$ ($P < 0.001$)

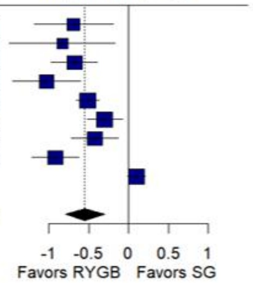

Study	MD	95%-CI	I2
Omitting Benaiges, 2024	-0.54	[-0.80; -0.27]	92.7%
Omitting Ceperuelo-Mallafré, 2019	-0.53	[-0.79; -0.27]	92.7%
Omitting Hofsø, 2019	-0.53	[-0.81; -0.26]	92.3%
Omitting Lee, 2011	-0.49	[-0.74; -0.25]	91.9%
Omitting Pajecki, 2023	-0.56	[-0.84; -0.28]	91.8%
Omitting Salminen, 2022	-0.58	[-0.86; -0.31]	92.9%
Omitting Schauer, 2012	-0.57	[-0.84; -0.29]	92.8%
Omitting Vix, 2013	-0.50	[-0.75; -0.24]	91.2%
Omitting Yan, 2021	-0.63	[-0.81; -0.44]	62.1%
**Random effects model**	**-0.55**	**[-0.79; -0.30]**	**91.9%**

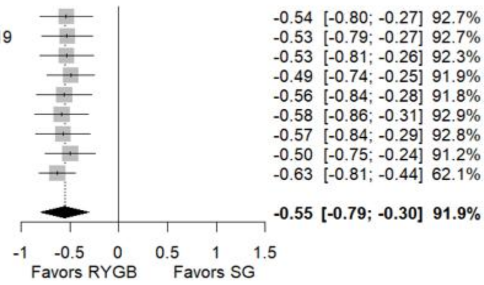

B. Between 1 and 10 years

Study	RYGB Mean	RYGB SD	RYGB Total	SG Mean	SG SD	SG Total	Weight	Mean Difference IV, Random, 95% CI
By-Band-Sleeve, 2025	2.40	0.74	461	2.89	0.97	419	19.0%	-0.49 [-0.60; -0.38]
Hauge, 2025	2.50	0.73	54	3.00	0.74	55	11.9%	-0.50 [-0.78; -0.22]
Murphy, 2022	2.90	0.90	50	2.90	0.90	53	9.4%	0.00 [-0.35; 0.35]
Pajecki, 2023	2.47	0.22	18	2.69	0.19	18	18.1%	-0.22 [-0.35; -0.09]
Peterli, 2018	2.62	0.94	53	3.01	0.94	68	9.7%	-0.39 [-0.73; -0.05]
Salminen, 2022	2.50	0.72	91	2.70	0.74	95	14.7%	-0.20 [-0.41; 0.01]
Schauer, 2017	2.42	0.92	49	2.98	1.10	47	7.8%	-0.56 [-0.97; -0.15]
Tang, 2016	2.40	0.70	38	3.10	0.80	34	9.4%	-0.70 [-1.05; -0.35]
**Total (95% CI)**			**814**			**789**	**100.0%**	**-0.37 [-0.51; -0.23]**

Heterogeneity: $Tau^2 = 0.0241$; $Chi^2 = 20.99$, df = 7 ($P = 0.0038$); $I^2 = 66.7\%$
Test for overall effect: $Z = -5.10$ ($P < 0.001$)

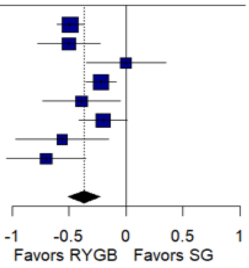

Study	MD	95%-CI	I2
Omitting By-Band-Sleeve, 2025	-0.34	[-0.50; -0.18]	57.1%
Omitting Hauge, 2025	-0.35	[-0.51; -0.19]	70.1%
Omitting Murphy, 2022	-0.40	[-0.54; -0.27]	63.6%
Omitting Pajecki, 2023	-0.40	[-0.56; -0.24]	58.7%
Omitting Salminen, 2022	-0.40	[-0.55; -0.24]	67.1%
Omitting Peterli, 2018	-0.37	[-0.53; -0.21]	71.4%
Omitting Schauer, 2017	-0.35	[-0.50; -0.20]	70.2%
Omitting Tang, 2016	-0.34	[-0.47; -0.20]	65.5%
**Random effects model**	**-0.37**	**[-0.51; -0.23]**	**66.7%**

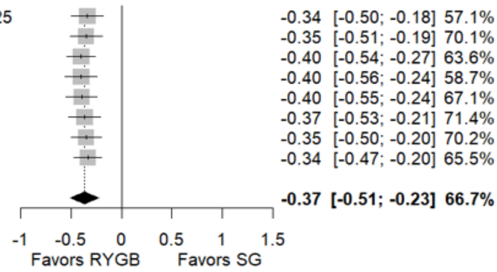

C. 10 years

Study	RYGB Mean	RYGB SD	RYGB Total	SG Mean	SG SD	SG Total	Weight	Mean Difference IV, Fixed, 95% CI
Kraljević, 2025	2.52	0.72	54	2.80	1.13	67	22.2%	-0.28 [-0.61; 0.05]
Salminen, 2022	2.30	0.70	86	2.40	0.49	96	77.8%	-0.10 [-0.28; 0.08]
**Total (95% CI)**			**140**			**163**	**100.0%**	**-0.14 [-0.30; 0.02]**

Heterogeneity: $Tau^2 = 0$; $Chi^2 = 0.88$, df = 1 ($P = 0.3485$); $I^2 = 0.0\%$
Test for overall effect: $Z = -1.75$ ($P = 0.079$)

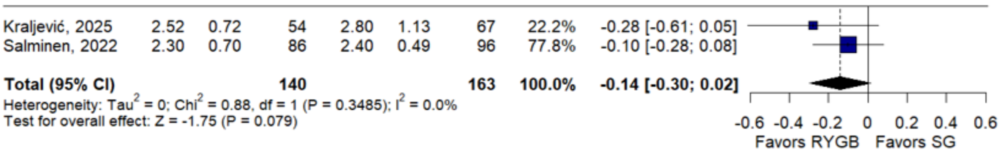

Abbreviations: RYGB: Roux-en-Y gastric bypass surgery; SG: Sleeve gastrectomy; CI: Confidence Interval; SD: Standard Deviation; IV: Inverse Variance; MD: Mean Difference.

© 2016 American Medical Association. All rights reserved.

Figure S3. Meta-analysis of mean differences in high-density lipoprotein cholesterol between RYGB and SG, including leave-one-out sensitivity analyses.

A. 1 year

Study	RYGB Mean	RYGB SD	RYGB Total	SG Mean	SG SD	SG Total	Weight	Mean Difference IV, Random, 95% CI
Benaiges, 2024	1.55	0.25	15	1.49	0.27	15	9.8%	0.06 [-0.13; 0.25]
Ceperuelo-Mallafré, 2019	1.33	0.23	15	1.46	0.30	15	9.6%	-0.13 [-0.32; 0.06]
Hofsø, 2019	1.20	0.22	54	1.26	0.22	55	14.4%	-0.06 [-0.14; 0.02]
Lee, 2011	1.28	0.20	30	1.18	0.20	30	13.6%	0.10 [-0.00; 0.20]
Pajecki, 2023	1.44	0.07	18	1.70	0.11	18	15.2%	-0.26 [-0.32; -0.20]
Schauer, 2012	1.52	0.47	50	1.45	0.35	49	10.8%	0.07 [-0.09; 0.23]
Vix, 2013	1.38	0.24	45	1.53	0.32	55	13.2%	-0.15 [-0.26; -0.04]
Yan, 2021	1.30	0.30	77	1.40	0.40	80	13.2%	-0.10 [-0.21; 0.01]
**Total (95% CI)**			**304**			**317**	**100.0%**	**-0.07 [-0.16; 0.03]**

Heterogeneity: $Tau^2 = 0.0136$; $Chi^2 = 50.12$, df = 7 ($P < 0.0001$); $I^2 = 86.0\%$
Test for overall effect: $Z = -1.42$ ($P = 0.157$)

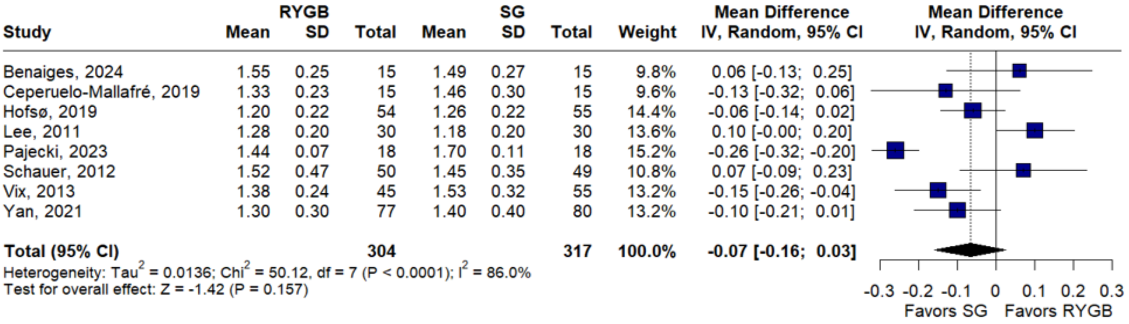

Study	MD	95%-CI	I2
Omitting Benaiges, 2024	-0.08	[-0.18; 0.02]	87.1%
Omitting Ceperuelo-Mallafré, 2019	-0.06	[-0.16; 0.04]	88.0%
Omitting Hofsø, 2019	-0.07	[-0.17; 0.04]	87.5%
Omitting Lee, 2011	-0.10	[-0.18; -0.01]	80.0%
Omitting Pajecki, 2023	-0.03	[-0.11; 0.04]	63.1%
Omitting Schauer, 2012	-0.08	[-0.18; 0.01]	86.6%
Omitting Vix, 2013	-0.05	[-0.16; 0.05]	87.9%
Omitting Yan, 2021	-0.06	[-0.17; 0.05]	88.0%
**Random effects model**	**-0.07**	**[-0.16; 0.03]**	**86.0%**

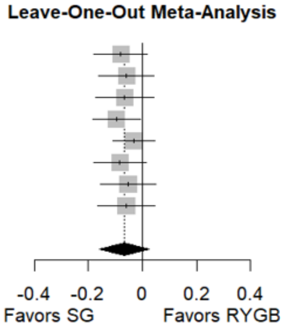

B. Between 1 and 10 years

Study	RYGB Mean	RYGB SD	RYGB Total	SG Mean	SG SD	SG Total	Weight	Mean Difference IV, Random, 95% CI
By-Band-Sleeve, 2025	1.67	0.50	461	1.57	0.47	419	29.8%	0.10 [0.04; 0.16]
Hauge, 2025	1.30	0.37	54	1.30	0.37	55	10.7%	0.00 [-0.14; 0.14]
Murphy, 2022	1.60	0.40	50	1.40	0.40	53	8.9%	0.20 [0.05; 0.35]
Pajecki, 2023	1.71	0.12	18	1.68	0.08	18	28.7%	0.03 [-0.04; 0.10]
Peterli, 2018	2.62	0.95	53	3.01	0.94	68	2.1%	-0.39 [-0.73; -0.05]
Schauer, 2017	1.55	0.52	49	1.48	0.43	47	6.2%	0.07 [-0.12; 0.26]
Tang, 2016	1.10	0.20	38	1.00	0.30	34	13.6%	0.10 [-0.02; 0.22]
**Total (95% CI)**			**723**			**694**	**100.0%**	**0.07 [0.02; 0.12]**

Heterogeneity: $Tau^2 = 0.0011$; $Chi^2 = 13.19$, df = 6 ($P = 0.0401$); $I^2 = 54.5\%$
Test for overall effect: $Z = 2.58$ ($P = 0.010$)

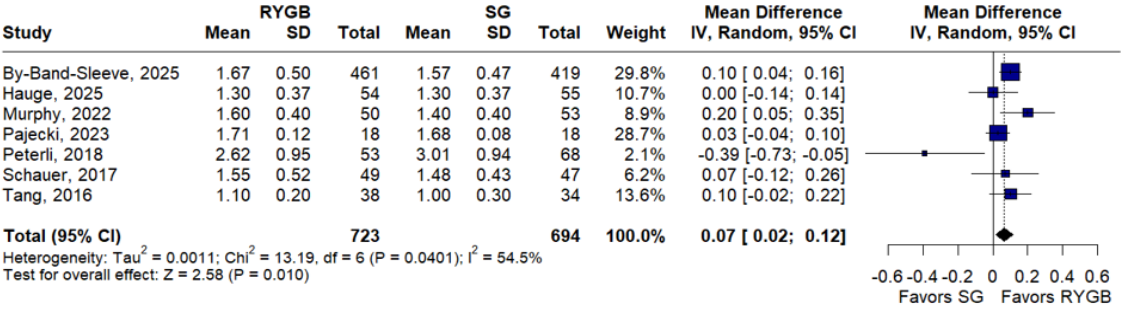

Study	MD	95%-CI	I2
Omitting By-Band-Sleeve, 2025	0.05	[-0.02; 0.12]	57.0%
Omitting Hauge, 2025	0.07	[0.02; 0.13]	59.1%
Omitting Murphy, 2022	0.05	[0.01; 0.10]	50.9%
Omitting Pajecki, 2023	0.09	[0.04; 0.13]	56.0%
Omitting Peterli, 2018	0.07	[0.03; 0.12]	18.5%
Omitting Schauer, 2017	0.06	[0.01; 0.12]	62.1%
Omitting Tang, 2016	0.06	[-0.01; 0.12]	61.2%
**Random effects model**	**0.07**	**[0.02; 0.12]**	**54.5%**

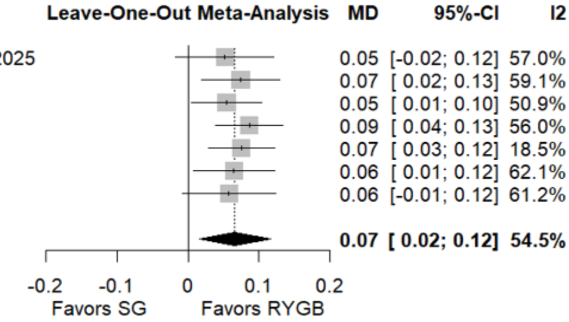

Abbreviations: RYGB: Roux-en-Y gastric bypass surgery; SG: Sleeve gastrectomy; CI: Confidence Interval; SD: Standard Deviation; IV: Inverse Variance; MD: Mean Difference.

© 2016 American Medical Association. All rights reserved.

Figure S4. Meta-analysis of mean differences in triglycerides between RYGB and SG, including leave-one-out sensitivity analyses.

A. 1 year

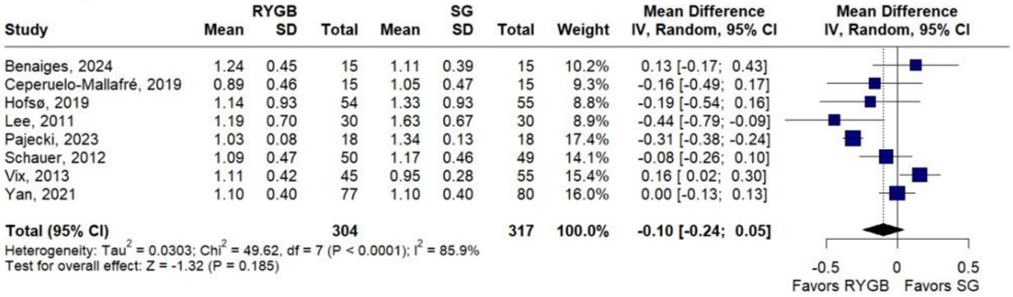

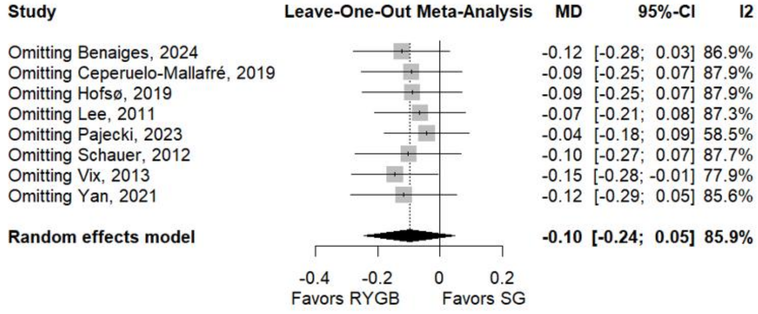

B. Between 1 and 10 years

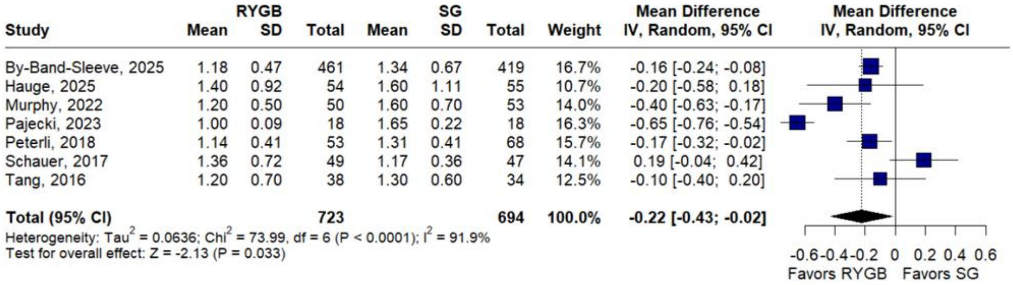

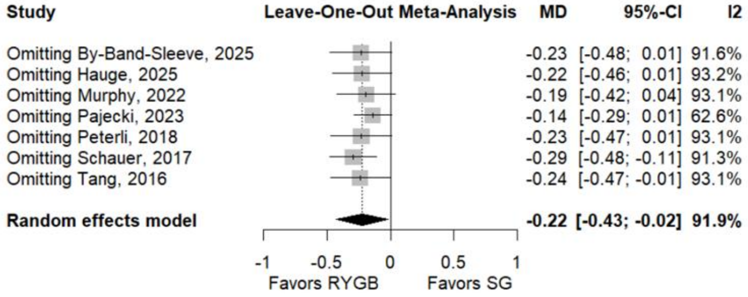

Abbreviations: RYGB: Roux-en-Y gastric bypass surgery; SG: Sleeve gastrectomy; CI: Confidence Interval; SD: Standard Deviation; IV: Inverse Variance; MD: Mean Difference.

© 2016 American Medical Association. All rights reserved.

Figure S5. Meta-analysis of mean differences in glycated hemoglobin between RYGB and SG, including leave-one-out sensitivity analyses.

A. 1 year

Study	RYGB Mean	RYGB SD	RYGB Total	SG Mean	SG SD	SG Total	Weight	Mean Difference IV, Random, 95% CI
Ceperuelo-Mallafré, 2019	5.18	0.65	15	6.08	1.06	15	7.8%	-0.90 [-1.53; -0.27]
Hofsø, 2019	5.90	1.10	54	6.20	1.11	55	10.2%	-0.30 [-0.71; 0.11]
Kalinowski, 2016	5.50	0.40	36	5.50	0.50	36	12.2%	0.00 [-0.21; 0.21]
Keidar, 2013	6.22	0.50	19	5.97	0.96	18	9.2%	0.25 [-0.25; 0.75]
Lee, 2011	5.70	0.50	30	7.20	1.50	30	8.5%	-1.50 [-2.07; -0.93]
Pajecki, 2023	5.62	0.20	18	5.93	0.24	18	12.7%	-0.31 [-0.45; -0.17]
Salminen, 2022	6.20	0.65	43	6.30	0.70	50	11.7%	-0.10 [-0.37; 0.17]
Schauer, 2012	6.40	0.90	50	6.60	1.00	49	10.6%	-0.20 [-0.58; 0.18]
Vix, 2013	5.40	2.33	45	5.51	2.59	55	5.1%	-0.11 [-1.08; 0.86]
Yan, 2021	5.20	0.80	77	5.30	0.70	80	12.0%	-0.10 [-0.34; 0.14]
**Total (95% CI)**			**387**			**406**	**100.0%**	**-0.30 [-0.57; -0.02]**

Heterogeneity: $Tau^2 = 0.1510$; $Chi^2 = 35.25$, df = 9 ($P < 0.0001$); $I^2 = 74.5\%$
Test for overall effect: Z = -2.09 (P = 0.036)

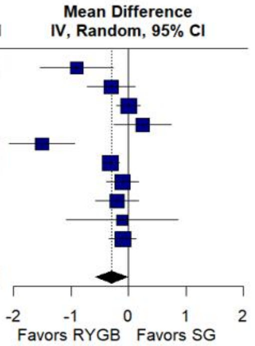

Study	MD	95%-CI	I2
Omitting Ceperuelo-Mallafré, 2019	-0.24	[-0.51; 0.03]	73.9%
Omitting Hofsø, 2019	-0.30	[-0.62; 0.02]	77.2%
Omitting Kalinowski, 2016	-0.34	[-0.65; -0.03]	73.5%
Omitting Keidar, 2013	-0.35	[-0.63; -0.06]	74.8%
Omitting Lee, 2011	-0.17	[-0.30; -0.04]	46.6%
Omitting Pajecki, 2023	-0.30	[-0.62; 0.03]	75.5%
Omitting Salminen, 2022	-0.33	[-0.64; -0.01]	76.8%
Omitting Schauer, 2012	-0.31	[-0.63; 0.01]	77.3%
Omitting Vix, 2013	-0.31	[-0.60; -0.01]	77.3%
Omitting Yan, 2021	-0.33	[-0.64; -0.01]	76.6%
**Random effects model**	**-0.30**	**[-0.57; -0.02]**	**74.5%**

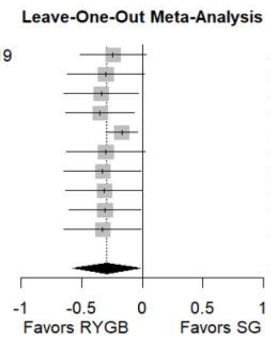

B. Between 1 and 10 years

Study	RYGB Mean	RYGB SD	RYGB Total	SG Mean	SG SD	SG Total	Weight	Mean Difference IV, Random, 95% CI
Biter, 2024	6.40	1.18	62	6.40	1.47	54	7.8%	0.00 [-0.49; 0.49]
By-Band-Sleeve, 2025	5.71	1.14	461	5.71	1.14	419	27.2%	0.00 [-0.15; 0.15]
Hauge, 2025	6.20	1.28	54	6.80	1.11	55	8.9%	-0.60 [-1.05; -0.15]
Pajecki, 2023	5.77	0.23	18	5.98	0.25	18	26.6%	-0.21 [-0.37; -0.05]
Peterli, 2018	5.90	0.52	28	6.20	0.99	26	9.7%	-0.30 [-0.73; 0.13]
Salminen, 2022	6.60	0.94	40	6.80	1.27	41	7.9%	-0.20 [-0.69; 0.29]
Schauer, 2017	7.30	1.50	49	7.40	1.60	47	5.3%	-0.10 [-0.72; 0.52]
Tang, 2016	6.40	1.60	38	6.00	0.60	34	6.5%	0.40 [-0.15; 0.95]
**Total (95% CI)**			**750**			**694**	**100.0%**	**-0.13 [-0.29; 0.02]**

Heterogeneity: $Tau^2 = 0.0169$; $Chi^2 = 12.55$, df = 7 ($P = 0.0838$); $I^2 = 44.2\%$
Test for overall effect: Z = -1.69 (P = 0.091)

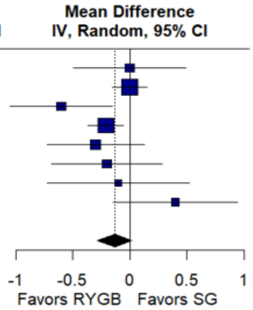

Study	MD	95%-CI	I2
Omitting By-Band-Sleeve, 2025	-0.20	[-0.32; -0.07]	30.4%
Omitting Hauge, 2025	-0.09	[-0.23; 0.05]	24.5%
Omitting Pajecki, 2023	-0.11	[-0.32; 0.09]	42.6%
Omitting Biter, 2024	-0.15	[-0.32; 0.03]	51.3%
Omitting Salminen, 2022	-0.13	[-0.30; 0.05]	51.8%
Omitting Peterli, 2018	-0.12	[-0.29; 0.06]	49.2%
Omitting Schauer, 2017	-0.14	[-0.31; 0.03]	52.2%
Omitting Tang, 2016	-0.17	[-0.32; -0.02]	33.4%
**Random effects model**	**-0.13**	**[-0.29; 0.02]**	**44.2%**

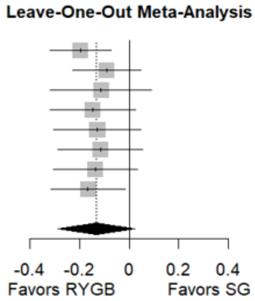

C. 10 years

Study	RYGB Mean	RYGB SD	RYGB Total	SG Mean	SG SD	SG Total	Weight	Mean Difference IV, Fixed, 95% CI
Kraljević, 2025	5.70	0.50	47	5.70	0.80	55	78.3%	0.00 [-0.26; 0.26]
Salminen, 2022	7.00	1.22	38	6.90	0.95	41	21.7%	0.10 [-0.38; 0.58]
**Total (95% CI)**			**85**			**96**	**100.0%**	**0.02 [-0.20; 0.25]**

Heterogeneity: $Tau^2 = 0$; $Chi^2 = 0.13$, df = 1 ($P = 0.7205$); $I^2 = 0.0\%$
Test for overall effect: Z = 0.19 (P = 0.851)

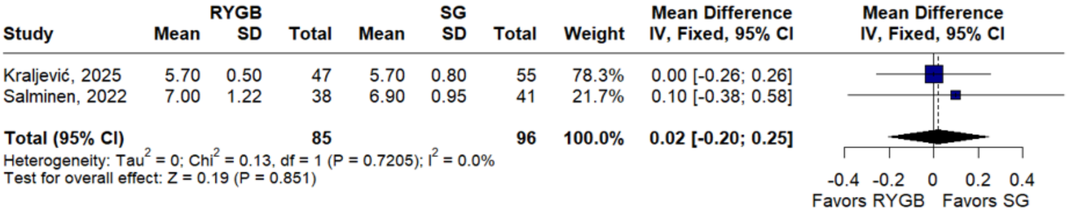

Abbreviations: RYGB: Roux-en-Y gastric bypass surgery; SG: Sleeve gastrectomy; CI: Confidence Interval; SD: Standard Deviation; IV: Inverse Variance; MD: Mean Difference.

© 2016 American Medical Association. All rights reserved.

Figure S6. Meta-analysis of mean differences in fasting glucose between RYGB and SG, including leave-one-out sensitivity analyses.

A. 1 year

Study	RYGB Mean	RYGB SD	RYGB Total	SG Mean	SG SD	SG Total	Weight	Mean Difference IV, Random, 95% CI
Ceperuelo-Mallafré, 2019	4.65	0.50	15	5.53	1.30	15	9.9%	-0.88 [-1.58; -0.18]
Hofsø, 2019	5.70	4.21	54	6.50	4.07	55	3.2%	-0.80 [-2.35; 0.75]
Kalinowski, 2016	4.50	0.70	36	4.50	0.70	36	17.0%	0.00 [-0.32; 0.32]
Lee, 2011	5.52	1.08	30	7.78	2.94	30	5.4%	-2.26 [-3.38; -1.14]
Salminen, 2022	5.90	1.14	43	6.40	1.23	50	13.8%	-0.50 [-0.98; -0.02]
Schauer, 2012	5.61	1.61	50	5.46	1.27	49	12.1%	0.15 [-0.42; 0.72]
Vix, 2013	4.86	0.60	45	4.72	0.40	55	19.3%	0.14 [-0.06; 0.34]
Yan, 2021	5.10	0.60	77	5.10	0.70	80	19.3%	0.00 [-0.20; 0.20]
**Total (95% CI)**			**350**			**370**	**100.0%**	**-0.26 [-0.56; 0.04]**

Heterogeneity: $Tau^2 = 0.1134$; $Chi^2 = 28.53$, df = 7 (P = 0.0002); $I^2 = 75.5\%$
Test for overall effect: Z = -1.68 (P = 0.094)

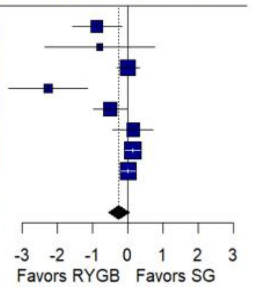

Study	MD	95%-CI	I2
Omitting Ceperuelo-Mallafré, 2019	-0.17	[-0.47; 0.12]	73.7%
Omitting Hofsø, 2019	-0.24	[-0.55; 0.07]	78.3%
Omitting Kalinowski, 2016	-0.35	[-0.72; 0.02]	78.9%
Omitting Lee, 2011	-0.09	[-0.31; 0.13]	54.5%
Omitting Salminen, 2022	-0.22	[-0.54; 0.11]	75.7%
Omitting Schauer, 2012	-0.33	[-0.67; 0.01]	78.7%
Omitting Vix, 2013	-0.39	[-0.78; -0.01]	75.3%
Omitting Yan, 2021	-0.39	[-0.80; 0.03]	78.9%
**Random effects model**	**-0.26**	**[-0.56; 0.04]**	**75.5%**

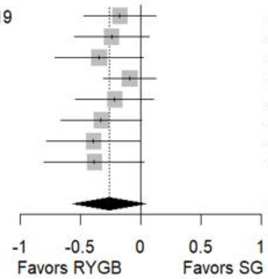

B. Between 1 and 10 years

Study	RYGB Mean	RYGB SD	RYGB Total	SG Mean	SG SD	SG Total	Weight	Mean Difference IV, Random, 95% CI
Biter, 2024	6.40	1.18	62	6.40	1.47	54	7.8%	0.00 [-0.49; 0.49]
By-Band-Sleeve, 2025	5.71	1.14	461	5.71	1.14	419	27.2%	0.00 [-0.15; 0.15]
Hauge, 2025	6.20	1.28	54	6.80	1.11	55	8.9%	-0.60 [-1.05; -0.15]
Pajecki, 2023	5.77	0.23	18	5.98	0.25	18	26.6%	-0.21 [-0.37; -0.05]
Peterli, 2018	5.90	0.52	28	6.20	0.99	26	9.7%	-0.30 [-0.73; 0.13]
Salminen, 2022	6.60	0.94	40	6.80	1.27	41	7.9%	-0.20 [-0.69; 0.29]
Schauer, 2017	7.30	1.50	49	7.40	1.60	47	5.3%	-0.10 [-0.72; 0.52]
Tang, 2016	6.40	1.60	38	6.00	0.60	34	6.5%	0.40 [-0.15; 0.95]
**Total (95% CI)**			**750**			**694**	**100.0%**	**-0.13 [-0.29; 0.02]**

Heterogeneity: $Tau^2 = 0.0169$; $Chi^2 = 12.55$, df = 7 (P = 0.0838); $I^2 = 44.2\%$
Test for overall effect: Z = -1.69 (P = 0.091)

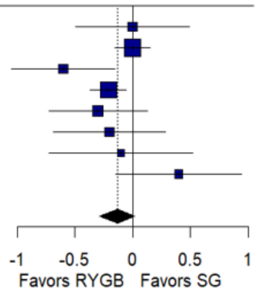

Study	MD	95%-CI	I2
Omitting By-Band-Sleeve, 2025	-0.20	[-0.32; -0.07]	30.4%
Omitting Hauge, 2025	-0.09	[-0.23; 0.05]	24.5%
Omitting Pajecki, 2023	-0.11	[-0.32; 0.09]	42.6%
Omitting Biter, 2024	-0.15	[-0.32; 0.03]	51.3%
Omitting Salminen, 2022	-0.13	[-0.30; 0.05]	51.8%
Omitting Peterli, 2018	-0.12	[-0.29; 0.06]	49.2%
Omitting Schauer, 2017	-0.14	[-0.31; 0.03]	52.2%
Omitting Tang, 2016	-0.17	[-0.32; -0.02]	33.4%
**Random effects model**	**-0.13**	**[-0.29; 0.02]**	**44.2%**

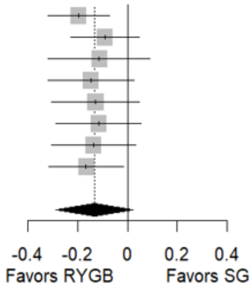

C. 10 years

Study	RYGB Mean	RYGB SD	RYGB Total	SG Mean	SG SD	SG Total	Weight	Mean Difference IV, Random, 95% CI
Kraljević, 2025	5.30	1.30	68	5.00	1.30	69	53.6%	0.30 [-0.14; 0.74]
Salminen, 2022	6.80	1.22	49	6.90	1.26	52	46.4%	-0.10 [-0.58; 0.38]
**Total (95% CI)**			**117**			**121**	**100.0%**	**0.11 [-0.28; 0.51]**

Heterogeneity: $Tau^2 = 0.0249$; $Chi^2 = 1.45$, df = 1 (P = 0.2283); $I^2 = 31.1\%$
Test for overall effect: Z = 0.57 (P = 0.566)

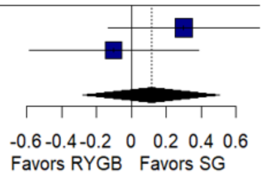

Abbreviations: RYGB: Roux-en-Y gastric bypass surgery; SG: Sleeve gastrectomy; CI: Confidence Interval; SD: Standard Deviation; IV: Inverse Variance; MD: Mean Difference.

© 2016 American Medical Association. All rights reserved.

Figure S7. Meta-analysis of mean differences in systolic blood pressure between RYGB and SG, including leave-one-out sensitivity analyses.

A. 1 year

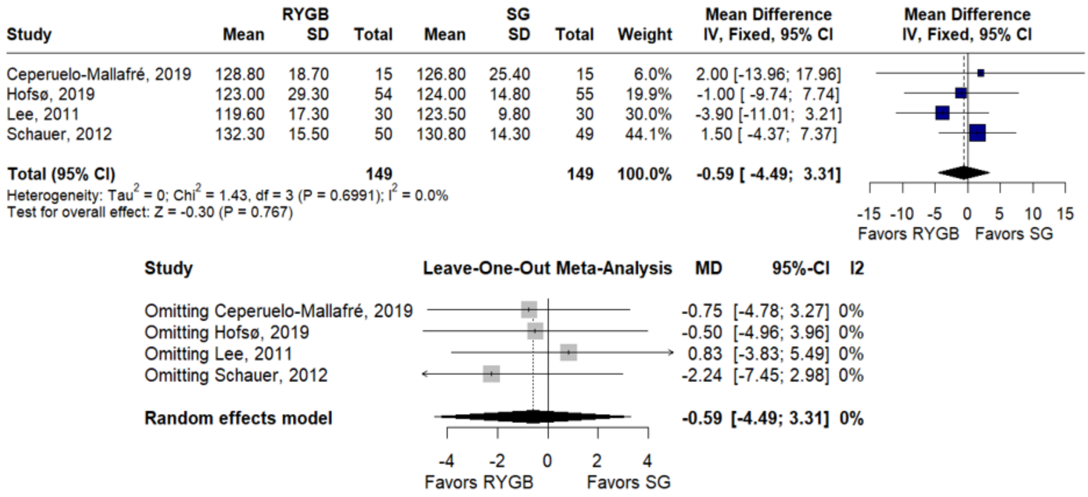

B. Between 1 and 10 years

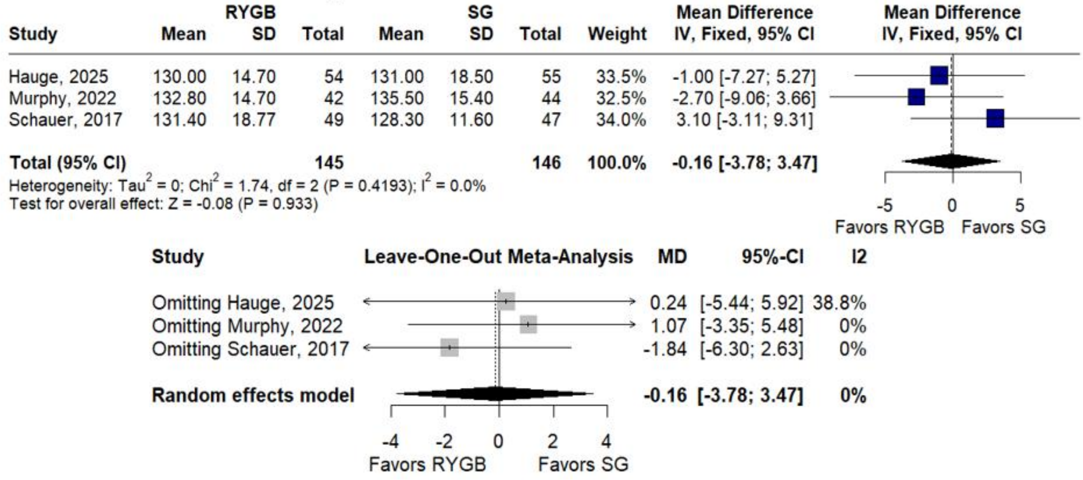

Abbreviations: RYGB: Roux-en-Y gastric bypass surgery; SG: Sleeve gastrectomy; CI: Confidence Interval; SD: Standard Deviation; IV: Inverse Variance; MD: Mean Difference.

© 2016 American Medical Association. All rights reserved.

Figure S8. Meta-analysis of mean differences in diastolic blood pressure between RYGB and SG, including leave-one-out sensitivity analyses.

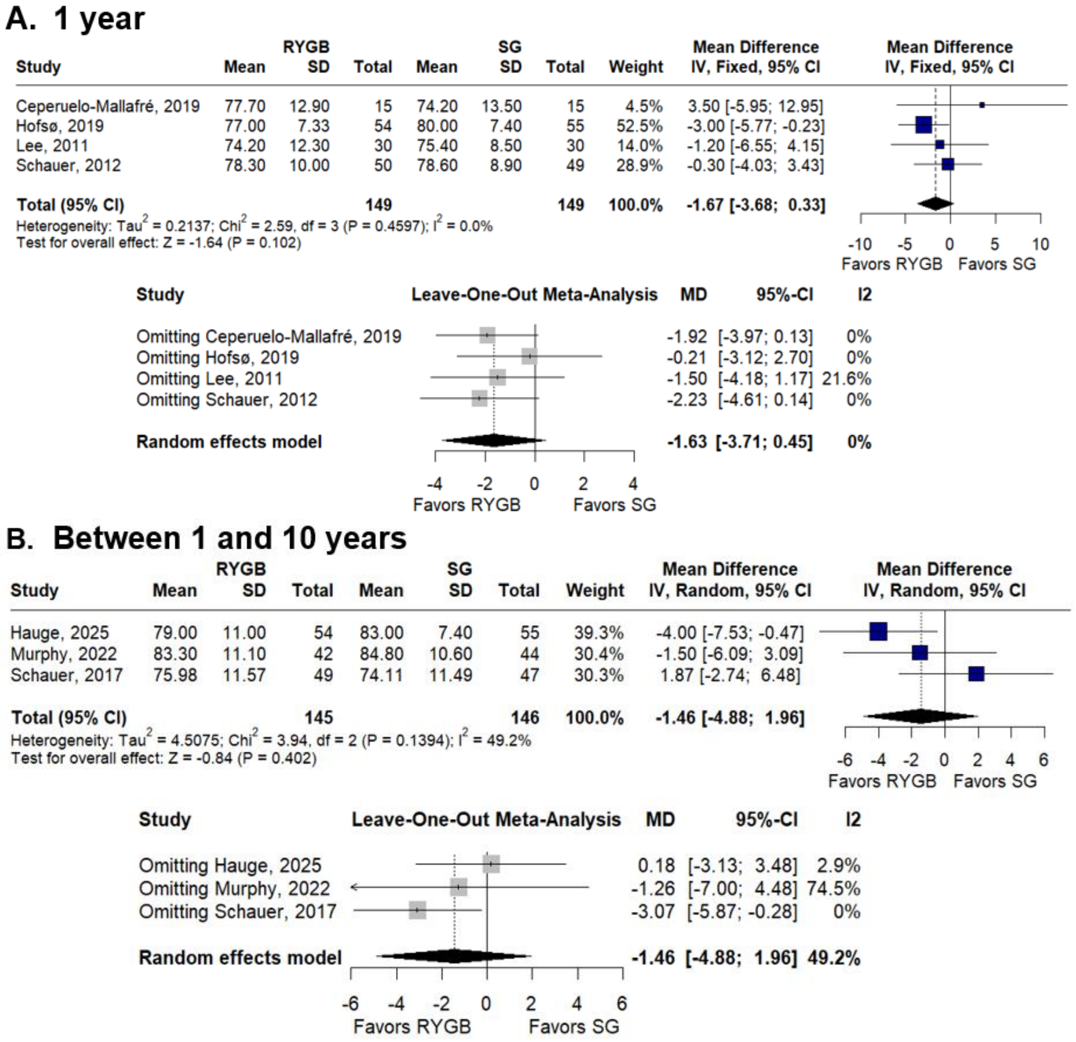

Abbreviations: RYGB: Roux-en-Y gastric bypass surgery; SG: Sleeve gastrectomy; CI: Confidence Interval; SD: Standard Deviation; IV: Inverse Variance; MD: Mean Difference.

Figure S9. Meta-analysis of mean differences in C-reactive protein between RYGB and SG, including leave-one-out sensitivity analyses.

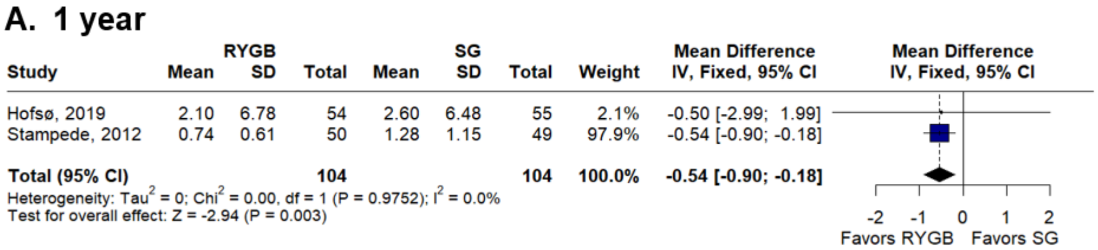

Abbreviations: RYGB: Roux-en-Y gastric bypass surgery; SG: Sleeve gastrectomy; CI: Confidence Interval; SD: Standard Deviation; IV: Inverse Variance; MD: Mean Difference.

© 2016 American Medical Association. All rights reserved.

Figure S10. Meta-analysis of risk ratios for dyslipidemia remission comparing RYGB and SG, including leave-one-out sensitivity analyses.

A. 1 year

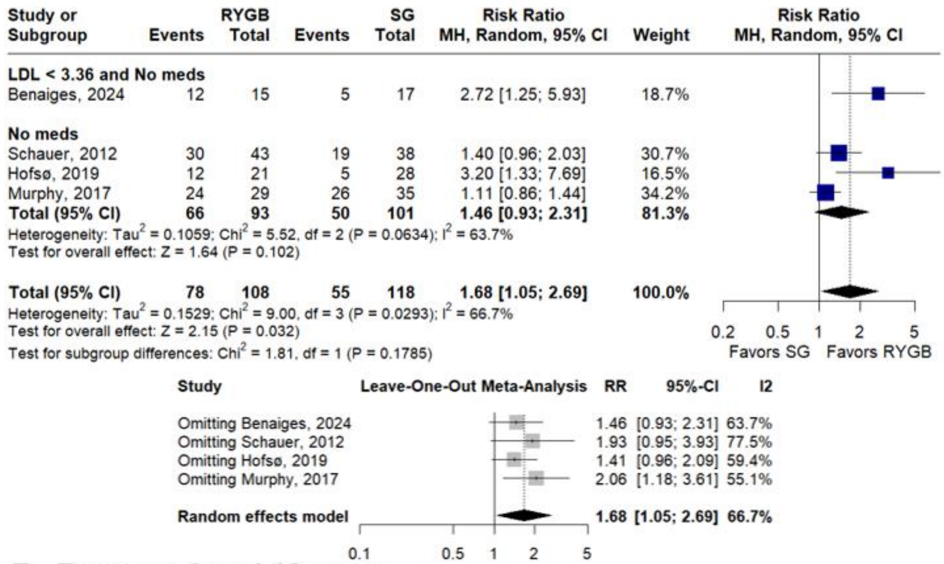

B. Between 1 and 10 years

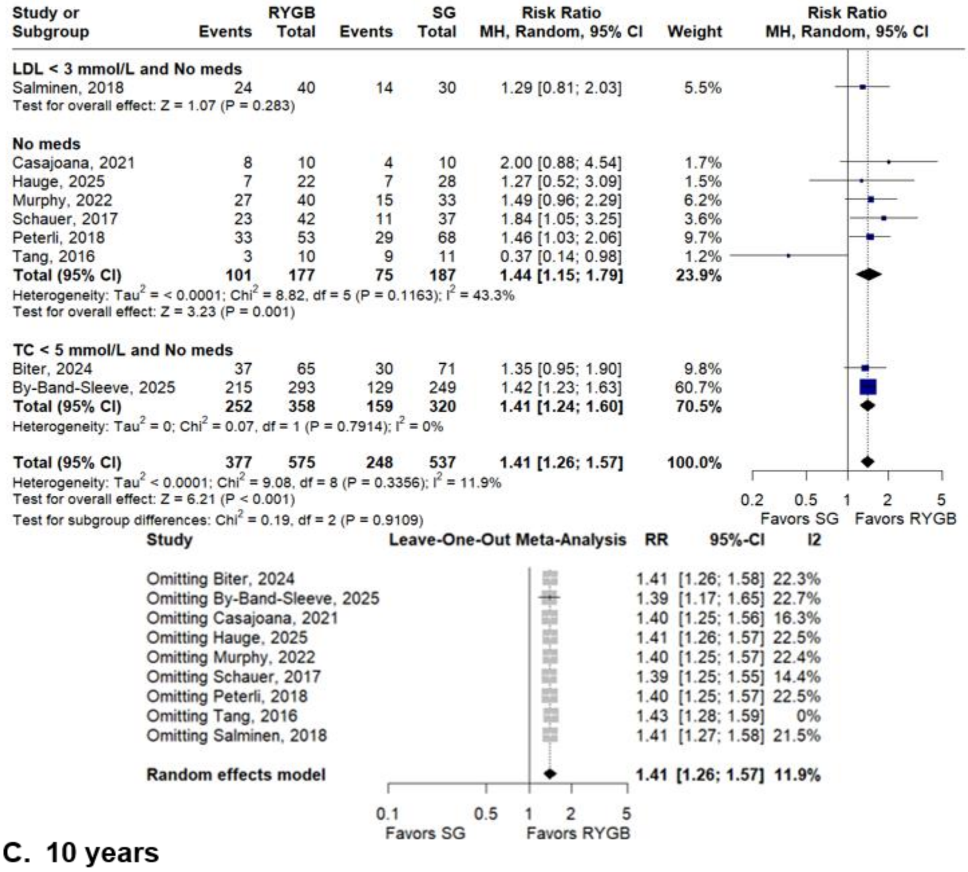

C. 10 years

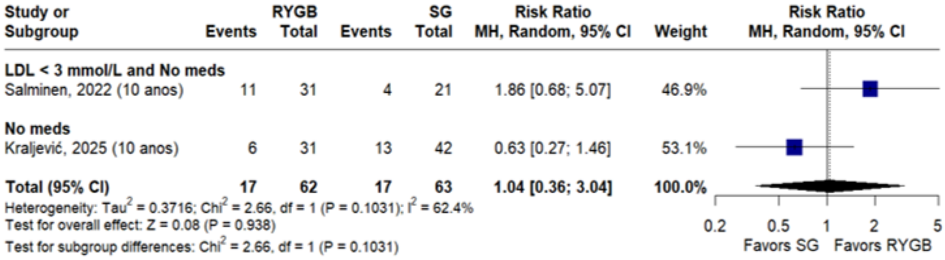

Abbreviations: RYGB: Roux-en-Y gastric bypass surgery; SG: Sleeve gastrectomy; CI: Confidence Interval; RR: Risk Ratio; MH: Mantel-Haenszel; LDL: Low-density lipoprotein cholesterol; TC: Total cholesterol; Meds: medications.

© 2016 American Medical Association. All rights reserved.

Figure S11. Meta-analysis of risk ratios for hypertension remission comparing RYGB and SG, including leave-one-out sensitivity analyses.

A. 1 year

Study	RYGB Events	RYGB Total	SG Events	SG Total	Weight	Risk Ratio MH, Fixed, 95% CI
Benaiges, 2024	5	8	6	10	10.3%	1.04 [0.50; 2.18]
Murphy, 2017	28	38	24	36	47.7%	1.11 [0.82; 1.49]
Schauer, 2012	23	39	20	33	42.0%	0.97 [0.67; 1.42]
**Total (95% CI)**	**56**	**85**	**50**	**79**	**100.0%**	**1.04 [0.83; 1.31]**

Heterogeneity: $Tau^2 = 0$; $Chi^2 = 0.27$, df = 2 (P = 0.8748); $I^2 = 0.0\%$
Test for overall effect: Z = 0.37 (P = 0.713)

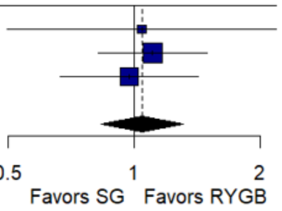

Study	RR	95%-CI	I2
Omitting Benaiges, 2024	1.05	[0.83; 1.33]	0%
Omitting Murphy, 2017	0.99	[0.70; 1.38]	0%
Omitting Schauer, 2012	1.10	[0.83; 1.45]	0%
**Random effects model**	**1.05**	**[0.84; 1.32]**	**0%**

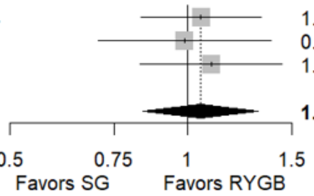

B. Between 1 and 10 years

Study	RYGB Events	RYGB Total	SG Events	SG Total	Weight	Risk Ratio MH, Random, 95% CI
Biter, 2024	52	103	46	111	18.9%	1.22 [0.91; 1.63]
By-Band-Sleeve, 2025	97	225	72	173	19.9%	1.04 [0.82; 1.31]
Casajoana, 2021	3	10	2	9	4.0%	1.35 [0.29; 6.34]
Hauge, 2025	14	37	16	20	15.6%	0.47 [0.30; 0.75]
Peterli, 2018	45	64	40	64	19.6%	1.12 [0.88; 1.44]
Salmien, 2022	37	73	20	68	16.2%	1.72 [1.12; 2.65]
Tang, 2016	3	16	5	12	5.8%	0.45 [0.13; 1.52]
**Total (95% CI)**	**251**	**528**	**201**	**457**	**100.0%**	**1.00 [0.71; 1.42]**

Heterogeneity: $Tau^2 = 0.1411$; $Chi^2 = 19.40$, df = 6 (P = 0.0035); $I^2 = 69.1\%$
Test for overall effect: Z = 0.03 (P = 0.978264)

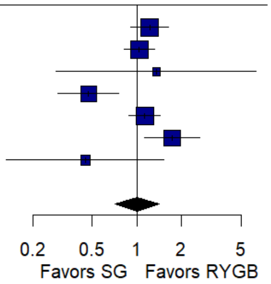

Study	RR	95%-CI	I2
Omitting Biter, 2024	0.95	[0.62; 1.46]	72.9%
Omitting By-Band-Sleeve, 2025	0.98	[0.63; 1.53]	74.1%
Omitting Casajoana, 2021	0.99	[0.69; 1.42]	74.1%
Omitting Hauge, 2025	1.15	[1.00; 1.32]	24.7%
Omitting Peterli, 2018	0.97	[0.62; 1.50]	73.9%
Omitting Salmien, 2022	0.91	[0.65; 1.29]	65.0%
Omitting Tang, 2016	1.06	[0.75; 1.50]	71.3%
**Random effects model**	**1.00**	**[0.71; 1.42]**	**69.1%**

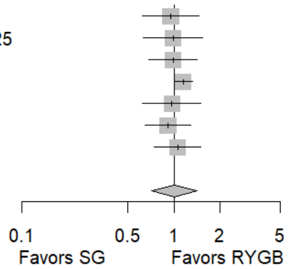

C. 10 years

Study	RYGB Events	RYGB Total	SG Events	SG Total	Weight	Risk Ratio MH, Fixed, 95% CI
Kraljević, 2025	20	41	21	40	78.5%	0.93 [0.60; 1.43]
Salminen, 2022	16	68	6	72	21.5%	2.82 [1.17; 6.79]
**Total (95% CI)**	**36**	**109**	**27**	**112**	**100.0%**	**1.34 [0.90; 1.98]**

Heterogeneity: $Tau^2 = 0.4933$; $Chi^2 = 4.97$, df = 1 (P = 0.0259); $I^2 = 79.9\%$
Test for overall effect: Z = 1.45 (P = 0.148)

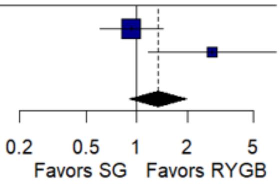

Abbreviations: RYGB: Roux-en-Y gastric bypass surgery; SG: Sleeve gastrectomy; CI: Confidence Interval; RR: Risk Ratio; MH: Mantel-Haenszel.

© 2016 American Medical Association. All rights reserved.

Figure S12. Meta-analysis of risk ratios for type 2 diabetes remission defined as HbA1c < 6%, comparing RYGB and SG, including leave-one-out sensitivity analyses.

A. 1 year

Study	RYGB Events	RYGB Total	SG Events	SG Total	Weight	Risk Ratio MH, Random, 95% CI
Benaiges, 2024	2	4	3	6	2.2%	1.00 [0.28; 3.54]
Casajoana, 2021	12	15	8	15	12.2%	1.50 [0.88; 2.57]
Hofsø, 2019	40	54	26	55	33.2%	1.57 [1.14; 2.16]
Kalinowski, 2016	9	14	6	12	7.5%	1.29 [0.65; 2.56]
Murphy, 2017	29	56	26	53	24.9%	1.06 [0.73; 1.53]
Pajecki, 2021	12	14	6	13	9.1%	1.86 [0.99; 3.47]
Schauer, 2012	21	50	13	49	10.9%	1.58 [0.90; 2.80]
**Total (95% CI)**	**125**	**207**	**88**	**203**	**100.0%**	**1.40 [1.16; 1.69]**

Heterogeneity: $Tau^2 = 0.0013$; $Chi^2 = 4.03$, df = 6 (P = 0.6721); $I^2 = 0.0\%$
Test for overall effect: Z = 3.49 (P = 0.000476)

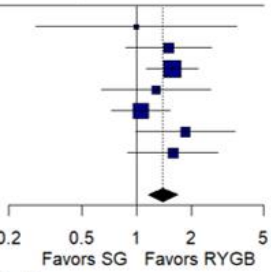

Study	RR	95%-CI	I2
Omitting Benaiges, 2024	1.41	[1.16; 1.71]	0%
Omitting Hofsø, 2019	1.33	[1.05; 1.67]	0%
Omitting Kalinowski, 2016	1.41	[1.15; 1.73]	0%
Omitting Murphy, 2017	1.54	[1.24; 1.91]	0%
Omitting Pajecki, 2021	1.36	[1.11; 1.67]	0%
Omitting Casajoana, 2021	1.39	[1.12; 1.72]	0%
Omitting Schauer, 2012	1.38	[1.12; 1.70]	0%
**Random effects model**	**1.40**	**[1.16; 1.69]**	**0%**

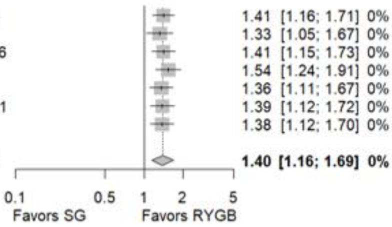

B. Between 1 and 10 years

Study	RYGB Events	RYGB Total	SG Events	SG Total	Weight	Risk Ratio MH, Random, 95% CI
Biter, 2024	35	62	30	54	19.8%	1.02 [0.74; 1.40]
Casajoana, 2021	7	14	3	14	5.6%	2.33 [0.75; 7.23]
Hauge, 2025	23	46	9	44	11.7%	2.44 [1.28; 4.68]
Murphy, 2022	27	56	18	58	15.8%	1.55 [0.97; 2.49]
Peterli, 2018	19	28	16	26	17.8%	1.10 [0.74; 1.64]
Salminen, 2018	5	41	10	40	6.9%	0.49 [0.18; 1.30]
Schauer, 2017	11	49	7	47	8.3%	1.51 [0.64; 3.56]
Tang, 2016	14	38	17	34	14.2%	0.74 [0.43; 1.26]
**Total (95% CI)**	**141**	**334**	**110**	**317**	**100.0%**	**1.20 [0.89; 1.62]**

Heterogeneity: $Tau^2 = 0.0925$; $Chi^2 = 14.81$, df = 7 (P = 0.0384); $I^2 = 52.8\%$
Test for overall effect: Z = 1.19 (P = 0.234398)

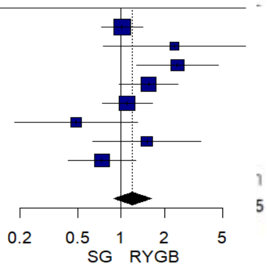

Study	RR 95%-CI	I2
Omitting Biter, 2024	1.25 [0.86; 1.83]	56.8%
Omitting Hauge, 2025	1.09 [0.87; 1.36]	35.4%
Omitting Peterli, 2018	1.23 [0.84; 1.80]	59.3%
Omitting Murphy, 2022	1.15 [0.81; 1.63]	53.9%
Omitting Salminen, 2018	1.27 [0.95; 1.71]	48.9%
Omitting Casajoana, 2021	1.15 [0.85; 1.57]	54.9%
Omitting Schauer, 2017	1.18 [0.84; 1.65]	58.4%
Omitting Tang, 2016	1.29 [0.96; 1.75]	48.8%
**Random effects model**	**1.20 [0.89; 1.62]**	**52.8%**

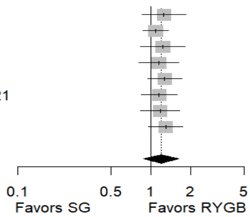

C. 10 years

Study or Subgroup	RYGB Events	RYGB Total	SG Events	SG Total	Risk Ratio MH, Random, 95% CI	Weight
**HbA1c < 6%**						
Kraljević, 2025	10	14	11	18	1.169 [0.712; 1.919]	51.8%
Grinlinton, 2025	15	49	7	40	1.749 [0.791; 3.870]	20.2%
**Total (95% CI)**	**25**	**63**	**18**	**58**	**1.309 [0.860; 1.993]**	**72.0%**
Heterogeneity: $Tau^2 = 0$; $Chi^2 = 0.71$, df = 1 (P = 0.3985); $I^2 = 0\%$						
Test for overall effect: Z = 1.25 (P = 0.210)						
**HbA1c < 6.5%**						
Salminen, 2022	13	39	11	42	1.273 [0.648; 2.498]	28.0%
**Total (95% CI)**	**38**	**102**	**29**	**100**	**1.299 [0.909; 1.855]**	**100.0%**

Heterogeneity: $Tau^2 = 0$; $Chi^2 = 0.72$, df = 2 (P = 0.6985); $I^2 = 0.0\%$
Test for overall effect: Z = 1.44 (P = 0.151)
Test for subgroup differences: $Chi^2 = 0.00$, df = 1 (P = 0.9452)

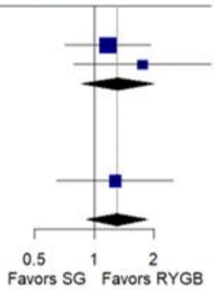

Study	RR	95%-CI	I2
Omitting Kraljević, 2025	1.45	[0.87; 2.43]	0%
Omitting Salminen, 2022	1.31	[0.86; 1.99]	0%
Omitting Grinlinton, 2025	1.20	[0.81; 1.80]	0%
**Random effects model**	**1.30**	**[0.91; 1.86]**	**0%**

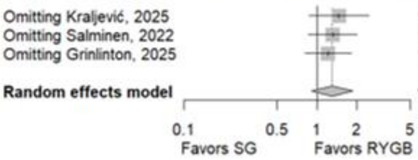

Abbreviations: RYGB: Roux-en-Y gastric bypass surgery; SG: Sleeve gastrectomy; CI: Confidence Interval; RR: Risk Ratio; MH: Mantel-Haenszel; HbA1C: Glycated hemoglobin.

© 2016 American Medical Association. All rights reserved.

Figure S13. Meta-analysis of risk ratios for type 2 diabetes remission defined as HbA1c < 6.5%, comparing RYGB and SG, including leave-one-out sensitivity analyses.

A. 1 year

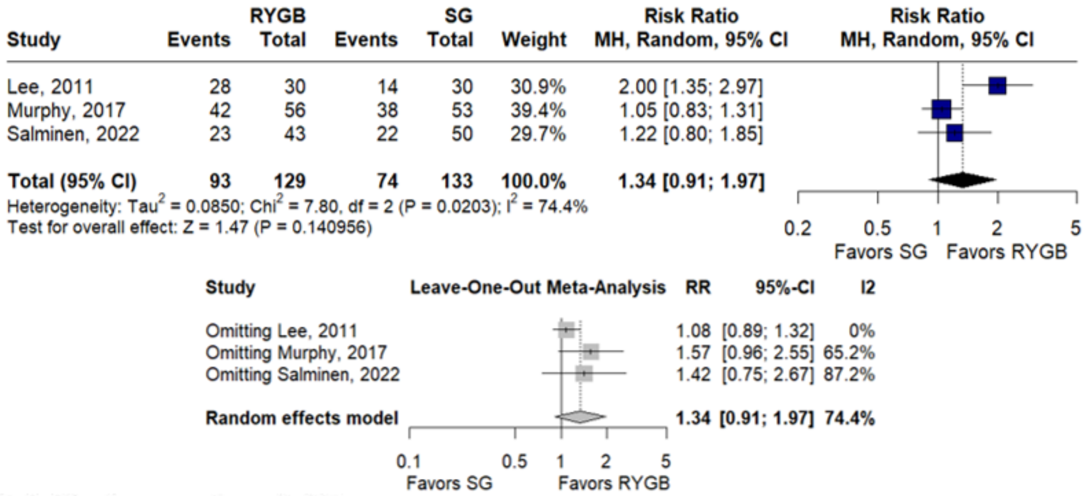

B. Between 1 and 10 years

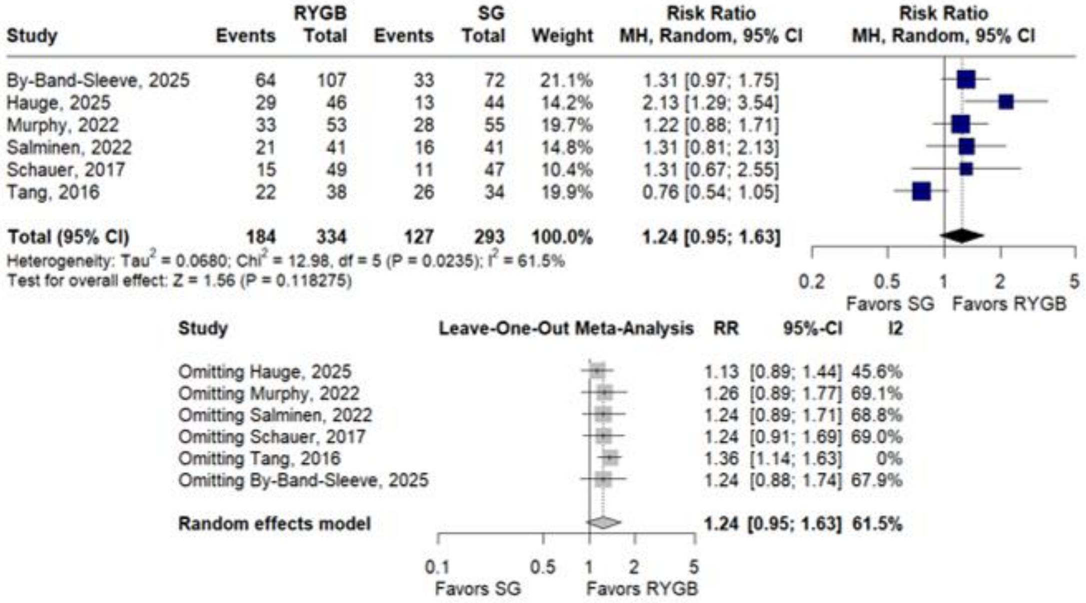

Abbreviations: RYGB: Roux-en-Y gastric bypass surgery; SG: Sleeve gastrectomy; CI: Confidence Interval; RR: Risk Ratio; MH: Mantel-Haenszel; HbA1C: Glycated hemoglobin.

© 2016 American Medical Association. All rights reserved.

Table S1. GRADE evidence profile for clinically important binary outcomes.

Certainty assessment							№ of patients		Effect		Certainty	Importance
№ of studies	Study design	Risk of bias	Inconsistency	Indirectness	Imprecision	Other considerations	RYGB	SG	Relative (95% CI)	Absolute (95% CI)		
**Hypertension remission 1y (follow-up: mean 1 year; assessed with: N (RR))**												
3[1-3]	randomised trials	not serious	not serious	not serious	serious[a]	none	56/85 (65.9%)	50/79 (63.3%)	**RR 1.04** (0.83 to 1.31)	**25 more per 1.000** (from 108 fewer to 196 more)	⊕⊕⊕◯ Moderate[a]	CRITICAL
**Hypertension remission between 1-10y (follow-up: mean 5 years; assessed with: N (RR))**												
7[4-10]	randomised trials	not serious	serious[b]	not serious	serious[c]	none	397/653 (60.8%)	315/581 (54.2%)	**RR 1.05** (0.82 to 1.34)	**27 more per 1.000** (from 98 fewer to 184 more)	⊕⊕◯◯ Low[b,c]	CRITICAL
**Hypertension remission 10y (follow-up: mean 10 years; assessed with: N (RR))**												
2[9,11]	randomised trials	not serious	not serious	not serious	very serious[d]	none	36/109 (33.0%)	27/112 (24.1%)	**RR 1.34** (0.90 to 1.98)	**82 more per 1.000** (from 24 fewer to 236 more)	⊕⊕◯◯ Low[d]	CRITICAL
**Dyslipidemia remission 1y (follow-up: mean 1 year; assessed with: N (RR))**												

© 2016 American Medical Association. All rights reserved.

Certainty assessment							№ of patients		Effect		Certainty	Importance
№ of studies	Study design	Risk of bias	Inconsistency	Indirectness	Imprecision	Other considerations	RYGB	SG	Relative (95% CI)	Absolute (95% CI)		
4[1-3,12]	randomised trials	not serious	serious[b]	not serious	not serious	none	78/108 (72.2%)	55/118 (46.6%)	**RR 1.68** (1.05 to 2.69)	**317 more per 1.000** (from 23 more to 788 more)	⊕⊕⊕◯ Moderate[b]	CRITICAL
**Dyslipidemia remission between 1-10y (follow-up: mean 5 years; assessed with: N (RR))**												
9[4-10,13,14]	randomised trials	not serious	not serious	not serious	not serious	none	377/575 (65.6%)	248/537 (46.2%)	**RR 1.27** (1.05 to 1.52)	**158 more per 1.000** (from 29 more to 305 more)	⊕⊕⊕⊕ High	CRITICAL
**Dyslipidemia remission 10y (follow-up: mean 10 years; assessed with: N (RR))**												
2[9,11]	randomised trials	not serious	serious[b]	not serious	very serious[d]	none	17/62 (27.4%)	17/63 (27.0%)	**RR 1.04** (0.36 to 3.04)	**11 more per 1.000** (from 173 fewer to 550 more)	⊕◯◯◯ Very low[b,d]	CRITICAL
**Diabetes remission (HbA1c < 6%) 1y (follow-up: mean 1 year; assessed with: N (RR))**												
7[1-3,6,12,15,18]	randomised trials	not serious	not serious	not serious	not serious	none	125/207 (60.4%)	88/203 (43.3%)	**RR 1.40** (1.16 to 1.69)	**173 more per 1.000** (from 69 more to 299 more)	⊕⊕⊕⊕ High	CRITICAL
**Diabetes remission (HbA1c < 6%) between 1-10y (follow-up: mean 5 years; assessed with: N (RR))**												

© 2016 American Medical Association. All rights reserved.

Certainty assessment							№ of patients		Effect		Certainty	Importance
№ of studies	Study design	Risk of bias	Inconsistency	Indirectness	Imprecision	Other considerations	RYGB	SG	Relative (95% CI)	Absolute (95% CI)		
8[4,6-10,13,14]	randomised trials	not serious	serious[b]	not serious	serious[a]	none	141/334 (42.2%)	110/317 (34.7%)	**RR 1.20** (0.89 to 1.62)	**69 more per 1.000** (from 38 fewer to 215 more)	⊕⊕◯◯ Low[a,b]	CRITICAL
**Diabetes remission (HbA1c < 6%) 10y (follow-up: mean 10 years; assessed with: N (RR))**												
3[9,11,19]	randomised trials	not serious	not serious	not serious	serious[e]	none	38/102 (37.3%)	29/100 (29.0%)	**RR 1.30** (0.91 to 1.86)	**87 more per 1.000** (from 26 fewer to 249 more)	⊕⊕⊕◯ Moderate[e]	CRITICAL
**Diabetes remission (HbA1c < 6.5%) 1y (follow-up: mean 1 year; assessed with: N (RR))**												
3[2,9,17]	randomised trials	not serious	serious[b]	not serious	serious[e]	none	93/129 (72.1%)	74/133 (55.6%)	**RR 1.34** (0.91 to 1.97)	**189 more per 1.000** (from 50 fewer to 540 more)	⊕⊕◯◯ Low[b,e]	CRITICAL
**Diabetes remission (HbA1c < 6.5%) between 1-10y (follow-up: mean 5 years; assessed with: N (RR))**												
6[5,7,9,10,13,14]	randomised trials	not serious	serious[b]	not serious	serious[a]	none	184/334 (55.1%)	127/293 (43.3%)	**RR 1.24** (0.95 to 1.63)	**104 more per 1.000** (from 22 fewer to 273 more)	⊕⊕◯◯ Low[a,b]	CRITICAL

RYGB: Roux-en-Y gastric bypass; **SG:** Sleeve Gastrectomy; **CI:** Confidence interval; **RR:** Risk ratio

© 2016 American Medical Association. All rights reserved.

Explanations

a. The imprecision is “Serious” because the 95% confidence interval crossing the line of no effect (RR = 1) and an insufficient total sample size, resulting in limited statistical power and substantial uncertainty regarding the true magnitude and direction of the effect.
b. The inconsistency is “Serious” (–1) because the heterogeneity observed was extremely high ($I^2 > 40\%$).
c. Imprecision for the “Hypertension remission between 1–10y” outcome was rated as “Serious” (–1) due to substantial between-study variability and uncertainty around the pooled effect estimate, because the 95% confidence interval crossing the line of no effect (RR = 1). The pooled analysis demonstrated considerable heterogeneity ($I^2 = 69\%$), and leave-one-out sensitivity analyses did not meaningfully reduce heterogeneity, except when omitting *Hauge et al., 2025*[7], which markedly lowered I^2 to approximately 21%. This finding suggests that the overall estimate is highly influenced by a single study and reflects unexplained inconsistency across studies, thereby limiting confidence in the precision of the effect estimate.
d. Imprecision was rated as “Very serious” (–2) due to the extremely limited evidence base, with only two contributing studies, and the presence of wide confidence intervals in both, each crossing the line of no effect (RR = 1). This reflects insufficient sample size and substantial uncertainty around the magnitude and direction of the effect.
e. Imprecision was rated as “Serious (–1)” due to the limited number of contributing studies (two) and confidence intervals crossing the line of no effect (RR = 1), indicating insufficient sample size and uncertainty in the long-term effect estimate.

© 2016 American Medical Association. All rights reserved.

References

1. Benaiges D, Goday A, Casajoana A, et al. Short-term effects of gastric bypass versus sleeve gastrectomy on high LDL cholesterol: The BASALTO randomized clinical trial. *Cardiovasc Diabetol*. 2024;23(1):205. Published 2024 Jun 15.
2. Murphy R, Clarke MG, Evennett NJ, et al. Laparoscopic Sleeve Gastrectomy Versus Banded Roux-en-Y Gastric Bypass for Diabetes and Obesity: a Prospective Randomised Double-Blind Trial. *Obes Surg*. 2017;28(2):293-302.
3. Schauer PR, Kashyap SR, Wolski K, et al. Bariatric surgery versus intensive medical therapy in obese patients with diabetes. *N Engl J Med*. 2012;366(17):1567-1576.
4. Biter LU, 't Hart JW, Noordman BJ, et al. Long-term effect of sleeve gastrectomy vs Roux-en-Y gastric bypass in people living with severe obesity: a phase III multicentre randomised controlled trial (SleeveBypass). *Lancet Reg Health Eur*. 2024;38:100836.
5. By-Band-Sleeve Collaborative Group. Roux-en-Y gastric bypass, adjustable gastric banding, or sleeve gastrectomy for severe obesity (By-Band-Sleeve): a multicentre, open label, three-group, randomised controlled trial. *Lancet Diabetes Endocrinol*. 2025;13(5):410-426.
6. Casajoana A, Guerrero-Pérez F, García Ruiz de Gordejuela A, et al. Role of Gastrointestinal Hormones as a Predictive Factor for Long-Term Diabetes Remission: Randomized Trial Comparing Metabolic Gastric Bypass, Sleeve Gastrectomy, and Greater Curvature Plication. *Obes Surg*. 2021;31(4):1733-1744.
7. Hauge JW, Borgeraas H, Birkeland KI, et al. Effect of gastric bypass versus sleeve gastrectomy on the remission of type 2 diabetes, weight loss, and cardiovascular risk factors at 5 years (Oseberg): secondary outcomes of a single-centre, triple-blind, randomised controlled trial. *Lancet Diabetes Endocrinol*. 2025;13(5):397-409.
8. Peterli R, Wölnerhanssen BK, Peters T, et al. Effect of Laparoscopic Sleeve Gastrectomy vs Laparoscopic Roux-en-Y Gastric Bypass on Weight Loss in Patients With Morbid Obesity: The SM-BOSS Randomized Clinical Trial. *JAMA*. 2018;319(3):255-265.
9. Salminen P, Grönroos S, Helmiö M, et al. Effect of Laparoscopic Sleeve Gastrectomy vs Roux-en-Y Gastric Bypass on Weight Loss, Comorbidities, and Reflux at 10 Years in Adult Patients With Obesity: The SLEEVEPASS Randomized Clinical Trial. *JAMA Surg*. 2022;157(8):656-666.
10. Tang Q, Sun Z, Zhang N, et al. Cost-Effectiveness of Bariatric Surgery for Type 2 Diabetes Mellitus: A Randomized Controlled Trial in China. *Medicine (Baltimore)*. 2016;95(20):e3522.
11. Kraljevic M, Süsstrunk J, Wölnerhanssen BK, et al. Long-Term Outcomes of Laparoscopic Roux-en-Y Gastric Bypass vs Laparoscopic Sleeve Gastrectomy for Obesity: The SM-BOSS Randomized Clinical Trial. *JAMA Surg*. 2025;160(4):369-377.
12. Hofsø D, Fatima F, Borgeraas H, et al. Gastric bypass versus sleeve gastrectomy in patients with type 2 diabetes (Oseberg): a single-centre, triple-blind, randomised controlled trial. *Lancet Diabetes Endocrinol*. 2019;7(12):912-924.
13. Murphy R, Plank LD, Clarke MG, et al. Effect of Banded Roux-en-Y Gastric Bypass Versus Sleeve Gastrectomy on Diabetes Remission at 5 Years Among Patients With Obesity and Type 2 Diabetes: A Blinded Randomized Clinical Trial. *Diabetes Care*. 2022;45(7):1503-1511.
14. Schauer PR, Bhatt DL, Kirwan JP, et al. Bariatric Surgery versus Intensive Medical Therapy for Diabetes - 5-Year Outcomes. *N Engl J Med*. 2017;376(7):641-651.
15. Kalinowski P, Paluszkiewicz R, Wróblewski T, et al. Ghrelin, leptin, and glycemic control after sleeve gastrectomy versus Roux-en-Y gastric bypass-results of a randomized clinical trial. *Surg Obes Relat Dis*. 2017;13(2):181-188.
16. Keidar A, Hershkop KJ, Marko L, et al. Roux-en-Y gastric bypass vs sleeve gastrectomy for obese patients with type 2 diabetes: a randomised trial. *Diabetologia*. 2013;56(9):1914-1918.

© 2016 American Medical Association. All rights reserved.

17. Lee WJ, Chong K, Ser KH, et al. Gastric bypass vs sleeve gastrectomy for type 2 diabetes mellitus: a randomized controlled trial. *Arch Surg*. 2011;146(2):143-148.

18. Pajecki D, Dantas ACB, Tustumi F, Kanaji AL, de Cleva R, Santo MA. Sleeve Gastrectomy Versus Roux-en-Y Gastric Bypass in the Elderly: 1-Year Preliminary Outcomes in a Randomized Trial (BASE Trial). *Obes Surg*. 2021;31(6):2359-2363.

19. Grinlinton ME, Patel P, Nair A, et al. Ten-Year Results of a Randomized Trial Comparing Banded Roux-en-Y Gastric Bypass to Sleeve Gastrectomy for Type 2 Diabetes and Weight Loss. Obes Surg. 2025;35(12):4949-4958.

© 2016 American Medical Association. All rights reserved.
